# Plant stress detection using multimodal imaging and machine learning: from leaf spectra to smartphone applications

**DOI:** 10.3389/fpls.2025.1670593

**Published:** 2026-01-02

**Authors:** Muhammad Shoaib, Sajid Ullah Khan, Hala AbdelHameed, Ayman Qahmash

**Affiliations:** 1 Department of Computer Science, CECOS University of IT and Emerging Sciences, Peshawar, Pakistan; 2 Department of Computer Science and Information Technology, Faculty of Information Technology, The University of Lahore, Lahore, Pakistan; 3 Informatics and Computer Systems Department, King Khalid University, Abha, Saudi Arabia; 4 Faculty of Computer and Artificial Intelligence, Fayoum University, Fayoum, Egypt; 5 Khaybar Applied College, Taibah University, Medina, Saudi Arabia

**Keywords:** field-deployable technologies, hyperspectral imaging, machine learning, multispectral imaging, plant stress detection, smartphone-based sensing

## Abstract

Plant leaf spectrophotometry has been used successfully as a means to detect stress, and it has been complemented by fluorescence analysis. This identification can be achieved in the ultraviolet (UV), visible (red, green, blue; RGB), near-infrared (NIR), and infrared (IR) spectral regions. Hyperspectral (measuring continuous wavelength bands) and multispectral (measuring discrete wavelength bands) imaging modalities can provide detailed information concerning the physiological well-being of plants, often diagnosing them at an earlier stage than visual or other more traditional biochemical assays. Because hyperspectral methods are highly sensitive and accurate, they cost a lot and produce vast quantities of data, which demand sophisticated computing software, and compared to multimedia, multispectral, and RGB cameras, they are less expensive and easier to carry but have reduced spectral resolution. Such methods are justified by thermal and fluorescence images revealing variations in the temperature and efficiency of photosynthesis of the leaves in response to stress. New digital imaging, thermal imaging, and optical filter technologies, and advancements in smartphone cameras have rendered low-cost, field-deployable platforms to monitor plant stress in real time feasible. Machine learning also supports these techniques by automating feature extraction, classification, and prediction to reduce the use of expensive instrumentation and human skill. But also problems like sensor calibration in a changing field, low model generalization across species and environments, and large, annotated datasets are needed. Beyond highlighting the relative strengths of the conventional and contemporary sensing approaches, the paper also examines the possibility of applying machine learning to multimodal images, as well as the growing impact of smartphone- based solutions in supplying inexpensive agricultural diagnostics. It concludes by overviewing the current limitations and limits to future research into scalable, cost-effective, and generalizable plant stress models.

## 1 Introduction

**Figure 1:**
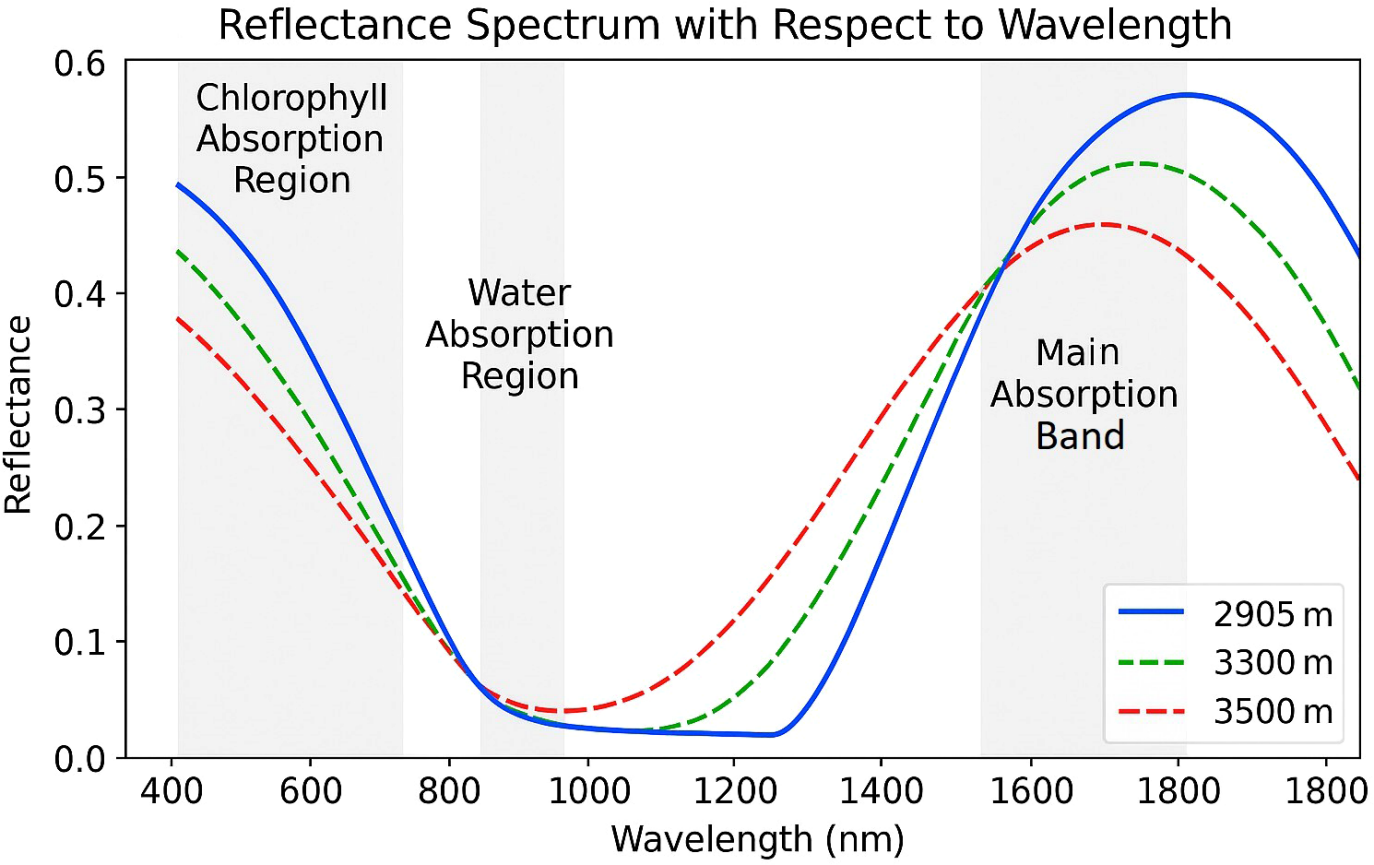
Quercus aquifolioides leaf reflectance spectra at various altitudes. The typical vegetation reflectance curve features attenuated reflectivity within the visible range due to pigment absorption, a pronounced red-edge shift into the near-infrared (NIR) with high NIR reflectance driven by internal leaf structure, and a distinct water absorption band near 1350–1450 nm. Shaded areas highlight chlorophyll absorption and water absorption regions, while the main absorption band indicates structural and hydration effects. Reflectance near 1300 nm is associated with the hydration dynamics of leaf tissues (Zhu et al., 2020).

**Figure 2:**
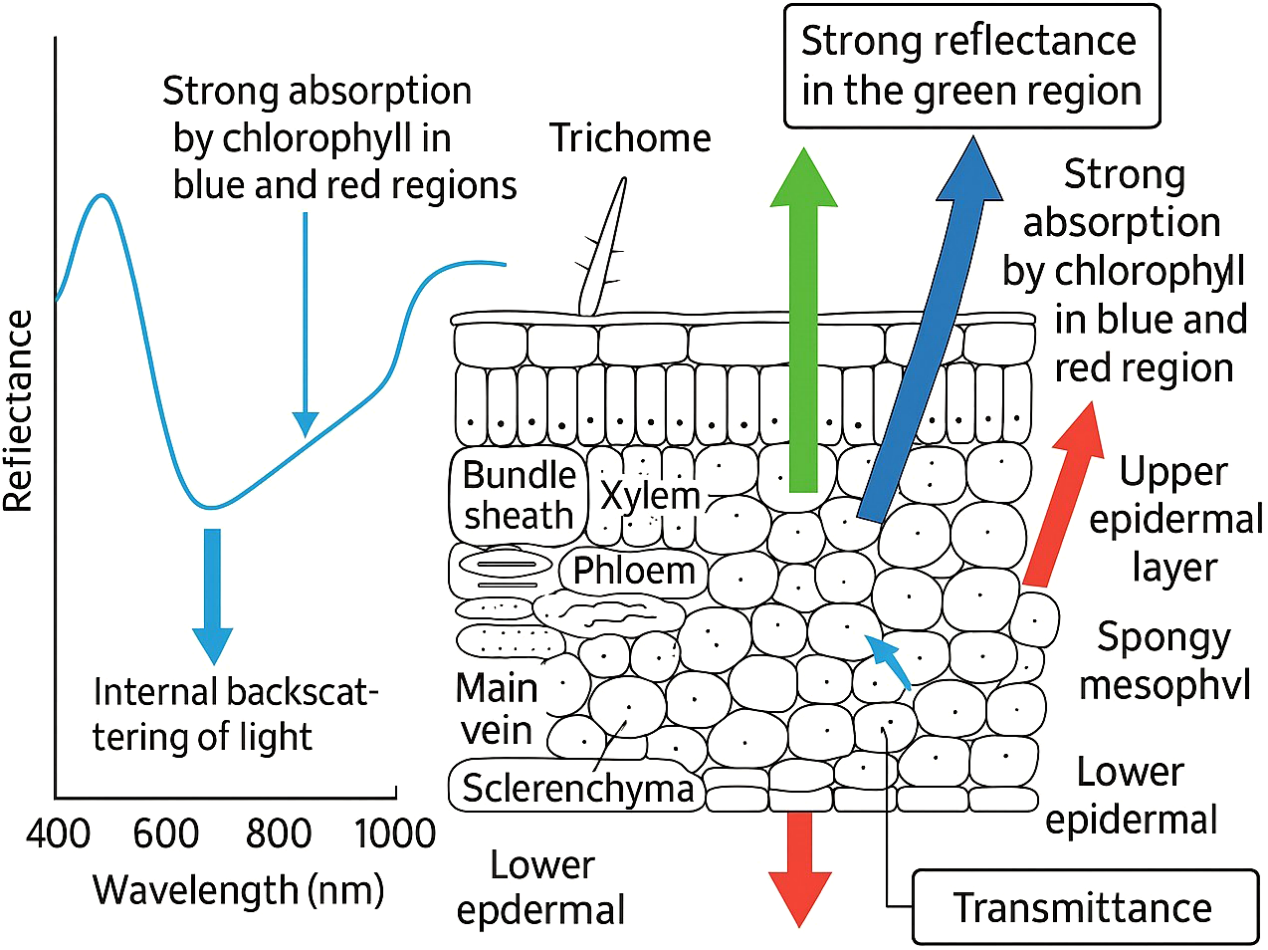
A cross-sectional view of a typical leaf and its corresponding spectral reflectance profile, illustrating labeled cell types and layers together with the fundamental interactions of light as it passes through these structures. The diagram highlights strong absorption by chlorophyll in the blue and red spectral regions, strong reflectance in the green region, internal backscattering within the mesophyll, and partial transmittance through the lower epidermis. These processes collectively explain the characteristic reflectance spectrum observed in healthy vegetation (Liew et al., 2008).

**Figure 3:**
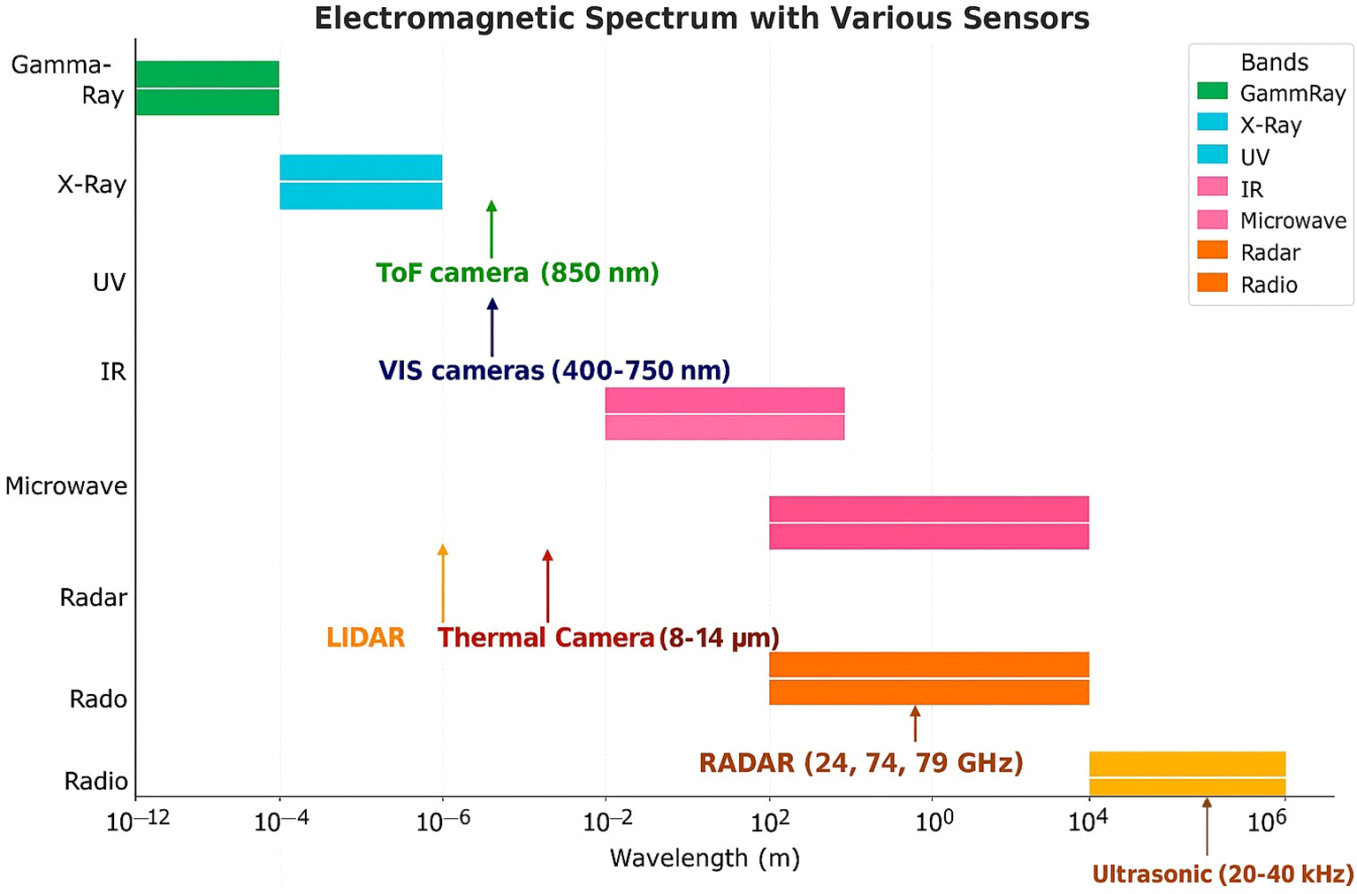
Electromagnetic spectrum segments employed by different optical sensors, including ultraviolet (UV), visible, and near-infrared (NIR) bands used for plant stress detection. Based on an adaptation of the work of (Rosique et al., 2019).

**Figure 4:**
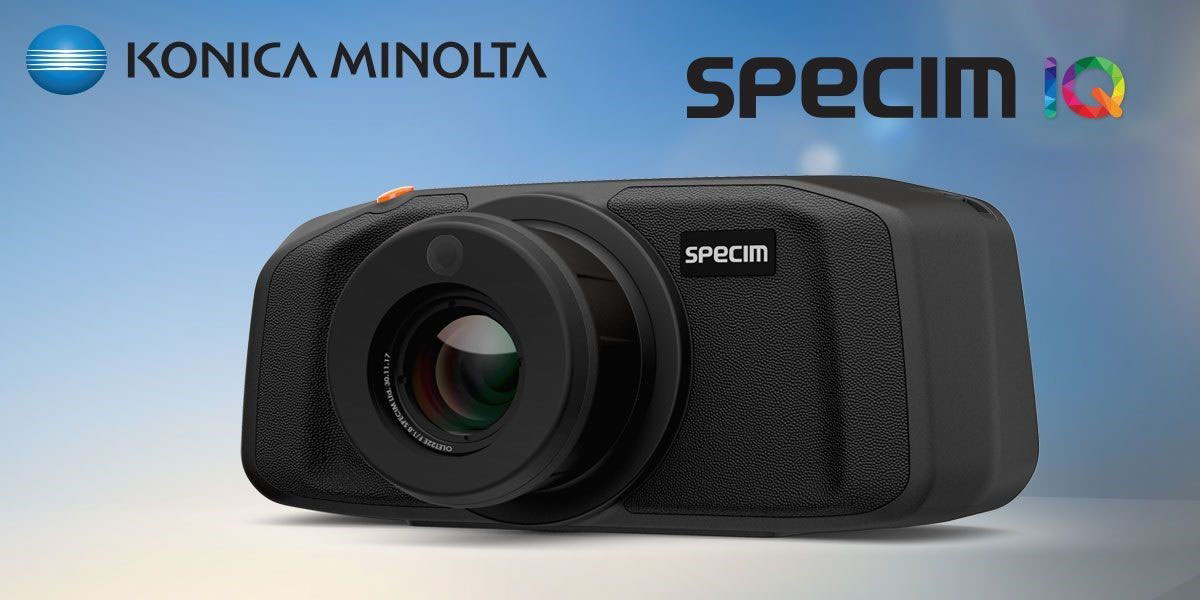
A compact hyperspectral camera (SpecimIQ). Adapted from (Behmann et al., 2018).

With the world population likely to grow to 9 billion by 2050 (Food and Agriculture Organization of the United Nations, 2017) (Food and Agriculture Organization of the United Nations, 2017), sustainable agricultural productivity has become more of a priority (Savary et al., 2017). This issue requires the incorporation of new approaches to improve crop production and reduce the negative impact of plant stressors like drought, nutrient deficiency, and disease (Savary et al., 2019). These diseases can rapidly disperse across regions through environmental vectors and human activities, with climate change intensifying their spread and altering their epidemiological patterns (Smith et al., 2021). Despite decades of research in plant physiology and pathology, early and accurate detection of plant stress remains a major challenge in sustainable agriculture. Conventional diagnostic techniques—though precise—are time-consuming, costly, and often infeasible at scale. As global agriculture transitions toward data-driven management systems, there is a pressing need for scalable, low-cost, and automated stress-detection frameworks that can operate in diverse field conditions. The convergence of multimodal imaging, including Red-Green-Blue (RGB), Near-Infrared (NIR), Short-Wave Infrared (SWIR), and Pulse- Amplitude-Modulated (PAM) fluorescence with machine learning, presents an unprecedented opportunity to continuously monitor crop health, extract physiological indicators from leaf reflectance spectra, and translate laboratory-level diagnostics into practical, smartphone-based applications. This paradigm shift provides the foundation for real-time decision-making and resilience against climate-induced stressors, thereby ensuring food security and resource optimization. Early detection of plant diseases is essential to prevent severe productivity losses and implement timely interventions. In this review, we focus on optical sensing pathways that enable such early detection and explicitly link spectral mechanisms to sensor choice and analysis throughout the manuscript (**Figures 1**–**4**; **Tables 1**–**6**).

**Table 1:** Optical methods used for the detection of plant stress.

Method	Wavelength(s)	Plant(s)	Stress type	Ref.
Hyperspectral Imaging	350–2500 nm	Maize, Barley, Wheat, Okra, Banana, Peanut	Drought, Rusts, Mildew, Salt Stress, Black Sigatoka, Leaf Spot	(Patel et al., 2014; Osco et al., 2020; Haralick et al., 1973; Pydipati et al., 2006; Krizhevsky et al., 2017; Tan and Le, 2019; Behmann et al., 2018; Chen et al., 2019; Zhang et al., 2023b)
Multispectral Spectroscopy	400–1100 nm	Maize, Tomato, Canola	Nutrient Deficiency, Drought Stress	(Kitic´ et al., 2019; Veys et al., 2017; Habibullah et al., 2020)
Multispectral Imaging	365–960 nm	Oilseed Rape, Tomato, Poinsettia, Wheat	Leaf Spot, Mold, Nutrient Deficiency, Rusts	(Veys et al., 2019; Fahrentrapp, 2019; Cardim Ferreira Lima et al., 2020; Adhikari et al., 2020; Bebronne et al., 2020)
RGB Imaging	RGB bands	Soybean, Black Gram, Potato, Basil	Nutrient Deficiency, Blight, Nitrogen Stress	(Naik et al., 2017; Watchareeruetai et al., 2018; Islam et al., 2017; Brambilla, 2020; Liu et al., 2024a)
Thermography	7.5–14 *µ*m	Grapes, Maize, Apple, Sesame, Wheat	Aspergillus, Drought Stress, Apple Scab	(Mastrodimos et al., 2019; Casari et al., 2019; Oerke et al., 2011; Khorsandi et al., 2018; Banerjee et al., 2020; Garcia et al., 2024; Wang et al., 2025)
Fluorescence Spectroscopy	337–650 nm	Passion Fruit, Maize, Tomato, Rapeseed, Grapefruit, Wheat	Drought, Nutrient Deficiency, Citrus Canker, Rust, Mildew	(Gomes et al., 2012; Kalaji et al., 2014, Kalaji et al., 2018; Burling et al., 2011; Saleem et al., 2020)
Fluorescence Imaging	340–760 nm	Barley, Grapevine, Sugar Beet, Soybean, Citrus, Cassava	Nutrient Deficiency, Leaf Spot, Herbicide Stress, Mosaic Virus	(Li et al., 2017; Konanz et al., 2014; Cen et al., 2017; Raji et al., 2015)

**Table 2:** Vegetation and disease indices: equations and applications.

Index Name	Equation	Application	Ref.
Normalized Difference Vegetation Index (NDVI)	$NDVI=\frac{R_{NIR}R_{RED}}{R_{NIR}+R_{RED}}$	Assessing plant growth dynamics	(Rouse et al., 1974)
Water Index (WI)	$WI\;=\frac{R_{900}}{R_{970}}$	Estimating water status	(Pen˜uelas et al., 1997)
Photochemical Reflectance Index (PRI)	$PRI=\frac{R_{570}-R_{531}}{R_{570}+R_{531}}$	Assessing photosynthetic efficiency	(Gamon et al., 1992)
Powdery Mildew Index (Wheat)	$PMI=(R_{515}-R_{698})-0.5R_{738}$	Detection of powdery mildew in wheat	(Huang et al., 2014)
Powdery Mildew Index (Sugar Beet)	$PMI=(R_{520}-R_{584})+R_{724}$	Detection of powdery mildew in sugar beet	(Mahlein et al., 2013)
Cercospora Leaf Spot Index (CLS)	$CLS=(R_{698}-R_{570})-R_{734}$	Detection of Cercospora leaf spot	(Mahlein et al., 2013)
Leaf Rust Disease Severity Index 1 (LRDSI1)	$LRDSI1=6.9\times \frac{R_{605}\;}{R_{455}}-1.2$	Assessing leaf rust severity	(Ashourloo et al, 2014)
Leaf Rust Disease Severity Index 2 (LRDSI2)	$LRDSI2=4.2\times \frac{R_{695}\;}{R_{455}}-038$	Evaluating wheat rust infection intensity	(Ashourloo et al., 2014)
Lemon Myrtle—Myrtle Rust Index (LMMR)	$LMMR=\frac{R_{545}xR_{555}\;}{R_{2195}}$	Detection of myrtle rust in lemon myrtle	(Heim et al., 2019)
Chlorophyll Carotenoid Index (CCI)	$CCI=\frac{R_{700}-R_{670}\;}{R_{700}+R_{670}}$	Monitoring carotenoid and chlorophyll content	(Zhang et al., 2023a)
Disease Stress Index (DSI)	$DSI=\frac{R_{680}-R_{720}\;}{R_{680}+R_{720}}$	Early detection of fungal diseases in cereals	(Kim et al., 2024)
Anthocyanin Reflectance Index (ARI)	$ARI=\frac{1\;}{R_{550}}-\frac{1}{R_{700}}$	Estimating anthocyanin concentration under stress	(Garcia et al., 2025b)

**Table 3:** Comparative assessment of optical monitoring approaches for plant stress detection.

Modality	Early sensitivity	Specificity	Cost (typ.)	Mobility/form	Calibration
RGB imaging	Low–Mod (visible symptoms/colour shifts; indirect for early biochemical)	Low–Mod (phenotype-level; lighting/background confounders)	Low (phone/camera)	High (phone, handheld, UAV)	Low–Mod (white balance, colour const.)
Multispectral imaging/ spectroscopy	Mod (pigment and water indices)	Mod (depends on band selection)	Low–Mod (handheld/UAV; phone add-ons)	High (handheld, UAV, phone modules)	Mod (radiometric panel; env. norm.)
Hyperspectral imaging	High (fine-grained biochemical/structural; early)	High (rich ctra enable discrimination/index discovery)	High (cam.+proc.; spectro moderate)	Mod (compact; lab common)	High (radiometry, illumination, stray light, co- reg)
Thermal imaging	Mod (detects water stress via Δ*T*)	Low (temperature not stress-specific)	Low–Mod (handheld/phone; sci-grade high)	High (handheld, UAV)	Mod (emissivity; ambient comp.; irradiance logs)
Fluorescence spectroscopy/ imaging	High (fast photosynthetic response)	Mod (multiple stressors alter fluorescence similarly)	Low–High (portable/phone to lab-grade PAM)	Mod–High (portable; phone modules)	Mod–High (dark adapt.; exc./emiss.)

*Sensitivity* reflects the ability to detect pre-symptomatic/weak signals; *specificity* reflects discrimination among distinct stressors (biotic vs. abiotic; nutrient vs. water). Cost is relative across modalities and vendor tiers. Suitability assumes adherence to the calibration practices indicated.

**Table 4:** (Part A): Comparative analysis of imaging modalities for plant stress detection.

Technique	Strengths	Weaknesses	Best-suited conditions
Hyperspectral imaging	Highest sensitivity; early detection of subtle biochemical/structural changes	Expensive high data volume; calibration/illumination demands	Controlled experiments; high-precision phenotyping; index discovery
Multispectral imaging	Affordable; portable; supports targeted indices	Lower spectral resolution; cross-environment calibration	Field monitoring; stress-specific indices; UAV/handheld
RGB imaging	Widely accessible; very low cost (smartphones)	Low specificity; lighting/weather sensitive	Rapid, low-cost field screening; extension tools
Thermal imaging	Fast water-stress detection; portable	Not stress-specific; environment-dependent	Drought detection; irrigation scheduling
Fluorescence imaging	Sensitive to photosynthetic efficiency	Needs controlled excitation; limited specificity	Early physiological stress; greenhouse/high-control settings

**Table 4:** (Part B): Comparative analysis of machine-learning approaches for plant stress detection.

Method	Strengths	Weaknesses	Best-suited conditions
SVM	Strong on small/spectral datasets; good generalization	Limited with complex image data; kernel tuning	Spectral classification; limited labels
Random Forest / Ensembles	Robust to noise; handles heterogeneous features; feature importance	Accuracy can plateau vs. deep learning	Mixed-feature datasets; rapid baselines
ANN (shallow)	Flexible nonlinear modeling	Overfitting risk; tuning burden	Moderate datasets; tabular+engineered features
CNN	Learns spatial features; top image accuracy	Large labeled data; high compute	Image-based stress detection; high-res imagery
Transformer models	Long-range/multimodal fusion potential	Very high compute; less interpretable	Large-scale multimodal sensing; fusion tasks

**Table 5:** Qualitative trade-offs among imaging modalities in field conditions.

Modality	Relative cost	Sensitivity (early)	Specificity	Throughput	Notes
HSI	$$$	High	High (biochemical)	Medium	High dimensionality; robust to confounders with proper calibration and normalization.
Multispectral	$$	Medium–High	Medium–High	High	Good compromise; robust indices; simpler calibration than HSI.
RGB	$	Low–Medium	Low–Medium	Very High	Extremely accessible; benefits greatly from transfer learning and color/illumination control.
Thermal (IR)	$–$$	Medium (water stress)	Medium	High	Sensitive to microclimate; requires reference panels and meteorological metadata.
Fluorescence	$$	High (photosystem)	High	Low–Medium	Excitation-dependent; powerful for early stress with proper protocols.

**Table 6:** ML/DL approaches for detection and quantification of plant diseases/stresses using diverse imaging techniques.

Purpose		Data type		Plant		Disease/stress condition		Algorithm(s) used in the literature		Accuracy (%)		Ref.	
Identification		Fluorescence imaging		Zucchini		Soft rot		SVM, ANN, Logistic Regression (fluorescence features)		90, 100, 60		(Pineda et al, 2017)	
Quantificatio		nRGB (Digital Camera)		Soybean		Iron deficiency chlorosis (IDC)		Colour-feature ML (SVM/LDA/RF/Elastic- Net family)		99.7–97.3		(Naik et al., 2017; Zhang et al., 2017; Bai et al., 2018)	
Identification		Hyperspectral		Wheat		Crown rot (Fusarium)		Hyperspectral ML (SVM/RF; band/index selection)		74.14–50.0		(Buster et al, 2023)	
Identification		RGB		Tulip		Tulip breaking disease (virus symptoms)		Classical ML on colour/texture (earlier); Faster R-CNN (later)		86.0		(Polder et al., 2010, Polder et al., 2019b)	
Identification		Hyperspectral		Potato		Potato virus Y (PVY)		Deep CNN/FCN on hyperspectral cubes		92.0		(Polder et al, 2019a)
Classification		RGB (Smartphone)		Wheat		Powdery Stripe rust mildew;		Field/mobile images with CNNs (C- DenseNet/ResNet); mobile apps for severity		88.89, 77.78		(Tang et al., 2023a; Mi et al., 2020a)
Identification		Fluorescence imaging		Zucchini		Soft rot		Same as first row (SVM/ANN/LogReg on fluorescence)		100, 90, 60		(Pineda et al, 2017)
Classification		RGB		Cucumber		Downy mildew; Powdery mildew; Anthracnose; Leaf spot		SVM, RF, deep CNN (transfer learning)		92.6–81.9		(Ma et al., 2018)
Classification		Hyperspectral		Sugar beet		Powdery mildew; Rust; Cercospora leaf spot		SVM on hyperspectral reflectance		86.42		(Rumpf et al, 2010)
Classification/Quantification		RGB (Database)		Wheat		Multiple (blotch, mildew, rust, smut, black chaff)		VGG-FCN-S / VGG- FCN-VD16 with deep MIL (WDD2017)		97.95–73.0		(Lu et al., 2017a)
Quantificatio		nRGB (Database)		Apple		Black rot (severity)		CNNs (VGG16, ResNet50) for severity levels		90.4, 80.0		(Wang et al., 2017; Zubler and Yoon, 2020)
Identification		Hyperspectral		Oil palm		Orange disease spotting		Red-edge indices + Neural Network/MLP		86.0		(Golhani et al., 2019; Zubler and Yoon, 2020)
Classification		RGB (Database)		Pomegranat		eLeaf spot; Blight; Rot		ANN on segmented colour/texture features		90.0		(Dhakate and Ingole, 2015)
Quantificatio		nRGB (Smartphone)		Coffee		Rust; Miner; Cercospora (severity)		Multi-task DCNN (VGG16/ResNet50); mobile app pipeline		86.51–82.94		(Esgario et al., 2020; Zubler and Yoon, 2020)
Identification		RGB		Apple; Coffee		Leaf diseases (multi- dataset)		Transfer-learning CNNs (MobileNet/ResNet with feature fusion)		99.79; 97.12		(Sujatha et al, 2025)
Classification		RGB		Potato		Drought stress		UAV-image CNN (e.g., DenseNet121 + Grad- CAM)		96.3		(Patra and Sahoo, 2024)
Classification		RGB (Multi- crop)		Multiple crops		Leaf diseases (multi- crop)		CNNs (AlexNet/GoogLeNet/VGG PlantVillage & field datasets)		80–99.2;		(Mohanty et al., 2016; Ferentinos, 2018)

Abiotic stressors, including water scarcity and nutrient imbalances, also significantly affect crop output and are expected to intensify due to changing environmental conditions, which increasingly challenge crop resilience and necessitate adaptive strategies (Shaw and Osborne, 2011; Liu et al., 2024b). Traditional detection methods such as PCR (Sui et al., 2017), ELISA, and flow cytometry (Andolfi et al., 2009) offer high specificity but often require specialized knowledge, time, and resources. Visual inspections, while simple, are subjective and prone to evaluator bias (McKenzie et al., 1984). In contrast, optical sensing technologies provide rapid and objective stress detection with growing utility due to improved sensor portability and resolution (Naik et al., 2017). Hyperspectral and multispectral imaging technologies enable detection of subtle physiological changes in plants before symptoms become visible. However, these imaging techniques generate complex and voluminous datasets that require advanced statistical and computational approaches for interpretation. To orient readers, **Figure 3** summarizes the spectral bands covered by the modalities reviewed, **Table 1** collates representative wavelength ranges/targets, and **Table 2** lists vegetation and disease indices that operationalize these signals.

Machine learning algorithms have shown great promise in automating the analysis of spectral data, enabling efficient identification of stressors by recognizing subtle patterns associated with plant health (Zhang et al., 2023c; Li et al., 2023; Garcia et al., 2025a). The primary objective of this review is to synthesize recent advancements in optical sensing technologies and machine learning approaches for detecting biotic and abiotic plant stresses. By comparing traditional diagnostic methods with modern imaging-based techniques, we aim to clarify their relative strengths, limitations, and practical applicability. In doing so, this review highlights the emerging role of portable and smartphone-based platforms in democratizing access to stress diagnostics and discusses how machine learning enables scalable, automated analysis. Ultimately, this study provides a comprehensive overview of the potential of these technologies for real-world deployment in sustainable agricultural management. To aid method selection, we include a comparative decision matrix (**Table 4a**) pairing sensing modalities with algorithm families and constraints and an evidence summary (**Table 6**) consolidating reported performances across tasks and datasets.

Section 2 grounds sensing in plant spectral physiology (**Figures 1**, **2**). Section 3 maps these mechanisms to concrete sensors and indices (anchored by **Figure 3** and **Tables 1**, **2**, plus example hardware in **Figure 4**), adds a field-suitability comparison (**Table 3**; Section 3.8), and introduces the decision matrix (**Table 4**; Section 3.9). Section 4 details preprocessing and learning methods, tying choices back to **Table 4** and summarizing outcomes in **Table 6**. Datasets from Red-Green-Blue (RGB), Near-Infrared (NIR), and Short- Wave Infrared (SWIR) imaging modalities were used to train models that differentiate between healthy and stressed plants. Section 5 synthesizes limitations, a 5–10 year roadmap, and deployment guidance.

### 1.1 Review methodology

To ensure comprehensive and reproducible coverage, we adopted a structured, PRISMA-style workflow tailored to a narrative/scoping review of optical sensing and ML for plant stress detection. Where possible, extracted study details are integrated directly into the main-text analysis and tables so that visual elements support the narrative rather than interrupt it.

#### 1.1.1 Databases and sources

Web of Science Core Collection, Scopus, PubMed, IEEE Xplore, and Google Scholar (first ∼200 results per query to limit noise). Preprints (e.g., arXiv) were consulted only when a peer-reviewed version was unavailable.

#### 1.1.2 Timeframe and language

January 2000–April 2025; English only.

#### 1.1.3 Inclusion criteria

Peer-reviewed empirical studies reporting (i) optical sensing/imaging of plants (leaf–canopy) and (ii) algorithmic analysis (classical ML or DL) or methodological innovations enabling stress detection/quantification. Greenhouse and field studies were eligible.

#### 1.1.4 Exclusion criteria

Non-plant or purely non-optical studies; non-English; editorials/commentaries; conference abstracts without full papers; theses; duplicates; reviews (used for background only); studies lacking key methodological details (e.g., undefined bands or validation scheme).

#### 1.1.5 Screening and selection

**Figure 5:**
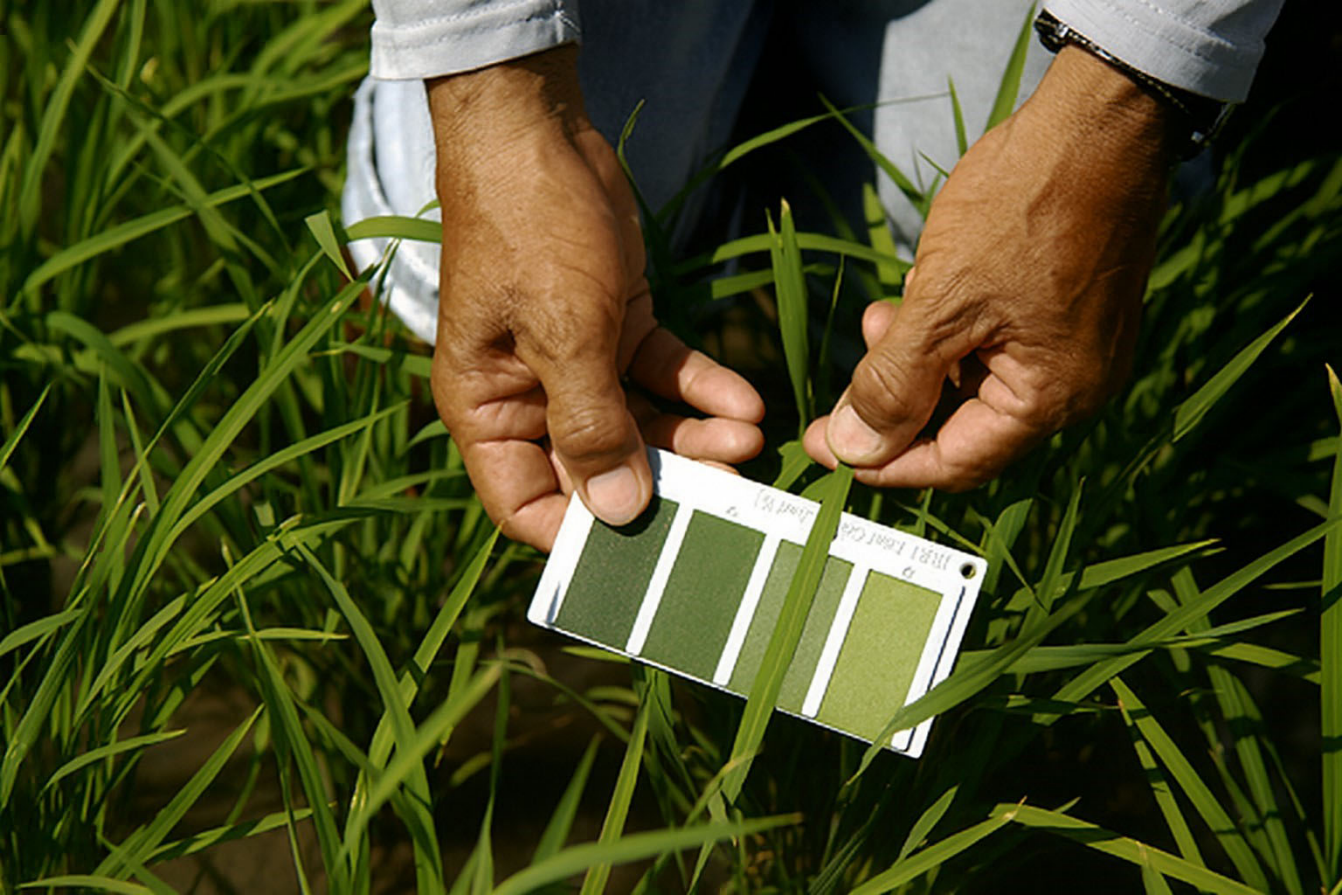
Use of smartphone-based imaging to assess rice leaf color for nitrogen deficiency detection. The image shows field application of a standard leaf color chart for visual comparison of greenness levels, which correspond to leaf nitrogen status. High-resolution enhancement improves visibility of color gradations and contextual detail for demonstration purposes (Casari et al., 2019).

Records were de-duplicated, then screened in two stages by two reviewers (MS, SUK): (1) title/abstract, (2) full text. Disagreements were resolved by discussion with a third author (AQ). Backward/forward snowballing captured additional eligible studies. Reasons for exclusion at the full-text stage were logged (e.g., inadequate ground truth, non-optical sensors). This ensured that items referenced in **Figures 1**–**4** and **Tables 1**–**6** adhere to consistent methodological standards. The use of smartphone-based imaging for assessing rice leaf color and detecting nitrogen deficiency is illustrated in **Figure 5** (Casari et al., 2019).

#### 1.1.6 Data extraction

For each study we charted plant species/crop; stress type (biotic/abiotic); environment (greenhouse/field); sensor/modality and bands; platform (handheld, smartphone, UAV); data volume; ground-truth/annotation; preprocessing; learning method and validation protocol; performance metrics; code/data availability; and key limitations. These data underpin comparative summaries in Sections 3–4 (e.g., modality attributes, indices, and ML trade-offs in **Table 4a** and the performance compendium in 6).

#### 1.1.7 Quality appraisal (fit-for-purpose)

Although not a meta-analysis, we applied a rubric to rate transparency and risk-of-bias across (i) sampling/replication, (ii) ground-truth rigor, (iii) validation design (cross-validation vs. held-out vs. external site/year), (iv) class-imbalance handling, (v) reporting completeness (confusion matrix/CI), and (vi) reproducibility (code/data). Ratings informed narrative weighting. We reflect these assessments when discussing limitations and benchmarks (see Sections 4.2.4 and 5.1).

#### 1.1.8 Methodological limitations

Heterogeneity in sensor configurations, environments, and reporting limited quantitative synthesis. While Google Scholar can introduce noise, multi-database coverage and snowballing mitigated omission risk. To maintain cohesiveness, we use cross-references so that figure/table callouts in the main text guide readers through the analysis; Section 2 follows with spectral foundations.

#### 1.1.9 Recency policy

Given the rapid pace of advances, we prioritized 2020–2025 studies as primary evidence and replaced older citations when updated studies were available, while retaining seminal references for historical context. This approach is reflected in Sections 3–4 (e.g., Patel et al., 2024a; Li et al., 2025a; Huang et al., 2023; Lei et al., 2024; Ammar et al., 2024; Kim et al., 2024, Zhang et al., 2023c, Liu et al., 2024b; Wang et al., 2025; Garcia et al., 2025a).

This structured workflow ensures methodological consistency across the reviewed literature and strengthens the reliability of the comparative analyses presented in Sections 3–5.

## 2 Spectral properties of plant tissues

The spectral reflectance of plant tissues is governed by their physiological characteristics and chemical composition, both of which are subject to change under stress conditions (Singh et al., 2023). Such stress-induced variations can alter a leaf’s reflectance profile, making spectral analysis a useful tool for stress detection (**Figure 1**). As shown in **Figure 1**, the canonical vegetation curve exhibits pigment-driven absorption in the visible range and a pronounced red-edge into the near-infrared (NIR); under stress, the red-edge typically shifts and green-band absorption decreases, consistent with these reports. Chlorophyll, a key pigment involved in photosynthesis, is particularly sensitive to stress. A reduction in chlorophyll content typically leads to increased reflectance near 700 nm (Zhu et al., 2020) and a concurrent decrease in reflectance in the 530–630 nm range (Lichtenthaler et al., 1996; Gitelson et al., 2002). These changes serve as indicators of compromised photosynthetic efficiency. In addition to chlorophyll, other pigments also modulate a plant’s reflectance properties (Vilfan et al., 2018; Li et al., 2022). These pigment-driven shifts underlie several indices summarized in **Table 2** (e.g., PRI, CCI, ARI), which operationalize the visible/red-edge signals described here and are later used with specific sensors in Section 3.

Leaf anatomical traits (**Figure 2**), such as the shape of epidermal cells (Bone et al., 1985), surface roughness, cuticle thickness (Grant et al., 1993), and trichome density (Ehleringer et al., 1976), also contribute to spectral behavior and are often modified by environmental stress. **Figure 2** locates the epidermis, palisade mesophyll, and spongy mesophyll layers whose thickness and internal air-space architecture chiefly govern visible absorption and NIR multiple scattering—clarifying how structural change appears in reflectance. For instance, ultraviolet radiation can induce changes in chlorophyll content and structural thickness, thereby affecting fluorescence emission (Bornman and Vogelmann, 1991). Pen˜uelas et al. Pen˜uelas et al. (1993) demonstrated that reflectance in the 950–970 nm range is linked to cell wall elasticity, which diminishes under drought stress (Pen˜uelas et al., 1993). Moreover, stomata—microscopic pores on the leaf surface—play a dual role: they regulate humidity and gas exchange while also acting as potential entry points for pathogens (Liew et al., 2008). Upon recognizing pathogen-associated molecular patterns (PAMPs), plants may induce stomatal closure as a defense strategy (Sawinski et al., 2013). This closure restricts transpiration and leads to a measurable increase in leaf surface temperature, which can be captured using infrared imaging. The structural and stomatal effects discussed here motivate thermal sensing (Section 3.4) and fluorescence methods (Sections 3.5–3.6), with **Table 2** providing complementary indices (e.g., WI for water status) that map onto these mechanisms.

Beyond structural and pigment-driven shifts, different stressors impart distinct and often diagnostic alterations to spectral reflectance profiles. For example, drought stress primarily reduces water absorption bands around 1450 nm and 1950 nm due to dehydration while also inducing a red-edge shift caused by cell wall shrinkage and reduced mesophyll scattering. Nutrient deficiencies, particularly nitrogen and phosphorus, result in decreased chlorophyll and protein content, leading to lowered absorption in the visible region and flatter red-edge transitions. Salinity stress disrupts mesophyll cell integrity and modifies carbohydrate–lignin composition, often elevating reflectance in the shortwave infrared (SWIR) domain (1300–2500 nm) due to ionic imbalances (de Lima et al., 2014). In contrast, pathogen infections frequently alter reflectance indirectly through changes in leaf surface structure, pigment degradation, and localized necrosis, which create heterogeneous reflectance ‘patches’ detectable in both visible and near-infrared regions (Mahlein et al., 2022). These lesion mosaics often dampen or shift the red edge and increase local variance in the 680–750,nm range, introducing high-frequency texture that can be leveraged by GLCM/entropy features or narrowband disease indices (e.g., PMI/CLS), thereby improving separability in hyperspectral and high-resolution multispectral imagery. These stress-specific spectral fingerprints directly inform the modality choices summarized in Sections 3 and the wavelength coverage illustrated in **Figure 3**.

Biochemical parameters such as protein, cellulose, starch, lignin, hemicellulose, and sugar content are also susceptible to environmental stressors, impacting spectral responses (Fourty et al., 1996; Mahlein et al., 2022). For example, salt stress can disrupt mesophyll cell integrity and alter the composition of structural carbohydrates such as polysaccharides and lignin (de Lima et al., 2014). Since water strongly absorbs light beyond 1300 nm in the infrared spectrum, variations in water content—another critical stress marker—significantly influence spectral characteristics (Allen and Richardson, 1968). These SWIR- and water-sensitive effects motivate the use of sensors covering 900–2500 nm (Section 3.1) and water-focused indices such as WI in **Table 2**.

### 2.1 Future research opportunities

While current research has focused primarily on the visible and NIR regions, underexplored spectral domains such as the short-wave infrared (SWIR, 1400–2500 nm) and terahertz ranges hold promise for detecting biochemical markers including lignin, secondary metabolites, and stress-induced volatiles. Additionally, coupling spectral measurements with omics data (e.g., metabolomics and proteomics) could reveal novel correlations between biochemical pathways and spectral features. Another important direction is developing universal spectral biomarkers capable of distinguishing overlapping stress responses, which would greatly improve field-level applications where multiple stressors often co-occur. Integrating these spectral insights with machine learning models (see Section 4) may enable earlier detection of plant stress and accelerate the translation of sensing technologies into precision agriculture. The spectral mechanisms summarized in this section directly inform sensor selection and data acquisition strategies in Section 3, where we align wavelength sensitivities with practical platforms (handheld, smartphone, UAV) and operational indices (**Tables 1**, **2**), and via the field-suitability comparison (**Table 3**; Section 3.8) prepare the ground for learning-based analysis in Section 4.

The mechanisms outlined above determine where diagnostically useful contrast lives in the spectrum and, therefore, which sensors are most informative. Pigment dynamics (chlorophyll/carotenoids) that shape the visible curve and red–edge favor RGB and visible–NIR multispectral cameras and indices such as PRI/CCI. Multiple scattering in the mesophyll that lifts NIR reflectance motivates narrow bands around the red–edge for structure-sensitive features. Water content and cell-wall elasticity leave strong imprints beyond 1300 nm, pointing to SWIR-capable hyperspectral/spectrometer systems and water indices (e.g., WI). Stomatal closure under stress elevates leaf temperature, motivating thermal infrared imaging. Energy-transfer changes in photosystems are captured by chlorophyll fluorescence spectroscopy/imaging. Section 3 operationalizes these links by mapping mechanisms to practical platforms, passbands, and field constraints.

## 3 Sensors and data collection

Building directly on the mechanisms in Section 2, we select sensors whose passbands intersect the diagnostic features they target—visible/red–edge bands for pigment and structural shifts, SWIR for water/biochemistry, thermal IR for stomatal/energy-balance responses, and fluorescence excitation/emission for photosystem kinetics (**Table 1**). These instruments capture reflectance data that may be visualized as images or expressed as spectral curves through spectroscopy. The effectiveness of each sensor largely depends on its sensitivity to specific regions of the plant’s reflectance profile, which are altered by both biotic and abiotic stress factors. Recent innovations in *in-situ* plant monitoring include a microneedle patch capable of detecting hydrogen peroxide levels in leaves, providing early biochemical indicators of plant stress before visual symptoms appear (Dong et al., 2025). The visible range of the electromagnetic spectrum has proven particularly responsive for evaluating plant health (Carter, 1993); however, stress indicators can also manifest across other regions of the spectrum. A pH-sensitive sensor based on chromatic covalent organic frameworks (COFs) has demonstrated the ability to detect drought-induced pH changes in plants up to 48 hours before visible stress symptoms (Strano et al., 2024). **Figure 3** situates each modality within the electromagnetic spectrum, while **Table 1** summarizes representative wavelength ranges, crops, stress targets, and references; throughout the section we tie these modalities back to the spectral cues in indices consolidated in **Table 2**.

The CropVoice platform leverages genetically modified plants to emit fluorescent signals under stress, which can be captured by drone, satellite, or tractor-mounted sensors to facilitate real-time agricultural monitoring (Aronov et al., 2024). These biochemical reporters complement reflectance-based cues and can be fused with optical measurements to improve early-warning specificity.

### 3.1 Hyperspectral imaging

Hyperspectral imaging captures fine pigment, water, and structural signatures across the VIS–NIR–SWIR spectrum, aligning with the red-edge and ¿1300 nm water bands highlighted in Section 2. This modality therefore provides the most detailed biochemical and structural information for early stress detection, albeit at higher cost and data complexity. Hyperspectral imaging is a fusion of imaging and spectroscopy to generate multidimensional data in which each pixel is represented in detail by its spectral characteristics at a large number of different specific wavelengths ( (Pandey et al., 2017)). This can give accurate discrimination of spatial regions and the detection of subtle spectral differences, which may otherwise be hidden in techniques that sum reflectance across whole leaves or images and may confound stressed and non-stressed tissues. This detail has made hyperspectral imaging a powerful tool in the agricultural research realm, namely, crop phenotyping and stress screening ( (Rumpf et al., 2010; Yang et al., 2019; Zovko et al., 2019)). It has been used to determine the reaction of the plants to different stressors, such as drought stress in banana [(Krizhevsky et al., 2017)] and maize [(Patel et al., 2014)], yellow rust in wheat [(Osco et al., 2020)], barley [(Fourty et al., 1996)], salt stress in okra (()**?**, and powdery mildew in several species [(Allen and Richardson, 1968)].

Hyperspectral imaging spectral range typically is 250 nm to 2500 nm, (including ultraviolet (UV) and near-infrared (NIR)) and visible and near-infrared are particularly important in identifying stress in plants ( (Deng et al., 2009)). This is despite the fact that much has been done on these bands, and new research is exploring other sections of the spectrum in an attempt to derive new stress indicators. As an example, hyperspectral imaging has just been used to measure salt stress in barley ( (Tan and Le, 2019)).

Hyperspectral data is commonly used in stress monitoring in the form of vegetation indices (VIs), which are calculated based on proportions or differences in reflectance across a particular range of wavelengths ( (Saleem et al., 2019; Mohanty et al., 2016; Lu et al., 2017b)). Spectral disease indices (SDIs) have also been designed to focus on individual plant diseases, in addition to VIs (Meng et al., 2020) (**Table 2**). Indicators of powdery mildew in sugar beet ( (Huang et al., 2014)) and wheat ( (Mahlein et al., 2013)) have been created as examples. The large amount of spectral data available with hyperspectral techniques makes possible the creation of new, stress-sensitive indices with the potential to improve the early detection of disease and stress ( (Pen˜uelas et al., 1997; Gamon et al., 1992)).

Despite its advantages in robustness and data richness, hyperspectral imaging has traditionally faced limitations related to the cost and size of equipment. Standard hyperspectral sensors are typically bulky and expensive, restricting their use in real-time field applications. However, technological advancements have led to the development of portable spectroradiometers and compact hyperspectral cameras (**Figure 4**), which, while offering a narrower spectral range, remain effective for targeted stress detection in real- time conditions (Balasundram et al., 2020; Behmann et al., 2018). Hyperspectral imaging is known to have limitations regarding equipment cost and size despite its benefits of robustness and richness of data. Conventional hyperspectral cameras are generally large and costly and can only be used in real-time field applications. However, as technology has been introduced, portable spectroradiometers and reduced-size hyperspectral cameras have been developed (**Figure 4**), albeit with a smaller spectral band, they can be applied to measure stress in real-time situations ( (Balasundram et al., 2020; Behmann et al., 2018)). Although spectroradiometers cannot capture hyperspectral images, they have been successfully used in various studies to detect stress symptoms such as peanut leaf spot (Chen et al., 2019) and powdery mildew in barley (Behmann et al., 2018).

#### 3.1.1 Limitations and practical considerations

Despite strong diagnostic capability, hyperspectral imaging faces practical constraints. Systems are costly (often several thousand USD), require careful radiometric calibration and controlled illumination to maintain accuracy, and generate high-volume data that demands advanced computational pipelines—complicating real-time field use. Portable spectroradiometers and compact cameras are emerging, but their narrower spectral coverage can limit versatility, and hardware cost remains a barrier to widespread adoption.

### 3.2 Multispectral imaging and spectroscopy

Multispectral imaging targets key wavelength regions, particularly around the green/red-edge and water absorption features that translate the pigment and structural cues described in Section 2 into practical sensing bands. By sampling only a few diagnostically relevant bands rather than the full spectrum, this approach operationalizes core spectral mechanisms at lower cost and with simpler deployment requirements.

Unlike hyperspectral methods, multispectral approaches collect data from a range of wavelengths rather than hundreds of specific or narrow wavelengths. Devices utilizing imaging or spectroscopic techniques can incorporate a few selected interest wavelengths. By using cameras or various other sensing equipment, data is collected to produce visual information in specific wavelength regions, a process known as multispectral imaging. Conversely, multispectral spectroscopy generates spectral data for specific wavebands.

Multispectral imaging and spectroscopy are used to identify plant stress, including leaf spot in oilseed rape ( (Veys et al., 2019)), gray mold in tomatoes ( (Fahrentrapp, 2019)), the insufficiency of nutrients ( (Cardim Ferreira Lima et al., 2020)), maize nitrogen stress ( (Kitic´ et al., 2019)), drought stress in tomatoes ( (Veys et al., 2017)), and canola nitrogen stress ( (Habibullah et al., 2020)). Although in general, multispectral techniques are cheaper than hyperspectral techniques because of the larger bandwidths employed, they lack the same amount of detailed information regarding the plant and its environment. Nevertheless, multispectral methods are suitable for developing customized devices due to the high mobility and versatility of these approaches. Specified spectral bands can be acquired at minimal cost with bandpass filters and cameras or other imaging devices. The cameras on smartphones have recently been upgraded to record near-infrared (NIR) wavelengths. To detect plant stress (Chung et al. (2018)), utilized an 800-nanometer high-pass filter that can be attached to a smartphone to capture both red and NIR images. In recent years, smartphone-based multispectral devices supported by deep learning have been shown to detect nutrient deficiencies in leafy vegetables in real time ( (Patel et al., 2024b)), and low-cost built-in cameras have been verified in sensing drought stress on maize fields ( (Li et al., 2025b)). Recent smartphone-integrated multispectral stacks have demonstrated reliable in-field inference for nutrient and water-stress diagnostics, combining clip-on optics with on-device models to deliver real-time decision support across small plots (Patel et al., 2024b; Garcia et al., 2025a; Li et al., 2025b).

#### 3.2.1 Limitations and practical considerations

Multispectral systems are more affordable and portable than hyperspectral platforms, yet lower spectral resolution can mask subtle physiological signals. Cross-environment calibration is nontrivial, and data quality can vary with filter characteristics and sensor type. Smartphone-based multispectral imaging is promising as a low-cost option, but heterogeneity in camera specifications and firmware across models complicates standardization for agriculture. Preprocessing and per-device calibration are key to the more stable accuracies reported in **Table 6** (see manuscript Section 4).

### 3.3 RGB imaging

RGB imaging, operating entirely within the visible spectrum, is inherently sensitive to pigment-related variations in reflectance. This sensitivity enables the derivation of color-based indices analogous to PRI and CCI, providing indirect proxies for chlorophyll and carotenoid dynamics discussed in Section 2. Although lacking NIR or SWIR information, RGB sensors offer valuable, low-cost access to stress-related color shifts. RGB imaging, also known as visible light imaging, employs sensors that capture image data in Red-Green-Blue (RGB) regions within the visible light spectrum, forming the basis of digital camera functionality. Specifically, blue light is detected in spectral bands that include blue (400–499 nm), green (500–549 nm) and red (550–750 nm), with peak responses at 475 nm, 520 nm and 650 nm, respectively (Deng et al., 2009). As such, RGB imaging is a subset of multispectral imaging. Typically, digital cameras and smartphones serve as the data acquisition tools for RGB imaging, whereas multispectral imaging requires more specialized instrumentation (Watchareeruetai et al., 2018).

One of the key positive aspects of RGB image capture lies in its cost-effectiveness and the sensor’s compact shape. Because smartphones already integrate RGB image sensors, they offer a practical and accessible platform for plant stress assessment. RGB imaging has been successfully applied to detect various plant stress conditions, iron chlorosis (a common issue in soybeans) (Naik et al., 2017), deficient nutrient levels in black gram (Xie et al., 2016), and biotic stresses, including early and late blight in potatoes and fungal diseases in wheat (Islam et al., 2017). Furthermore, the ubiquity of smartphones, combined with their onboard processing capabilities, allows for the rapid analysis and interpretation of imaging data with minimal technical expertise required. This democratizes the use of plant stress monitoring tools and facilitates real-time assessments in field conditions (Tao et al., 2020; Naik et al., 2017; Chen et al., 2024; Li et al., 2025a). The use of smartphone-based imaging for assessing rice leaf color and detecting nitrogen deficiency is illustrated in **Figure 5** (Casari et al., 2019). RGB lacks SWIR sensitivity but can proxy pigment dynamics underlying indices such as CCI/ARI in **Table 2**; pairing RGB with learned features (Section 4.2) often recovers much of the diagnostic signal at minimal hardware cost.

However, RGB data acquisition is susceptible to variability introduced by ambient brightness, external environment, daytime hours, and differences in camera spectral response. Such factors can affect image quality and reliability (Mattupalli et al., 2018). In agricultural field scenarios, variations in sunlight due to weather and seasonal changes are particularly influential. To mitigate these issues, machine- learning algorithms and advanced image-processing techniques are often used to improve the accuracy and robustness of RGB-based stress detection systems—for example, color constancy and illumination normalization (gray-card/white-balance calibration), shadow removal, and leaf background segmentation, followed by feature learning with transfer learning and data augmentation. Coupled with per-device calibration and simple domain-adaptation steps across sites/sensors, these pipelines yield more stable predictions under variable field conditions and support rapid, on-device inference (Naik et al., 2017).

#### 3.3.1 Limitations and practical considerations

RGB imaging is highly accessible, but measurements are sensitive to ambient illumination, solar angle, weather, and sensor response—all of which degrade reproducibility. Differences between smartphone and dedicated camera sensors further affect reliability. Moreover, RGB lacks direct sensitivity to many biochemical markers, limiting diagnostic specificity. Consequently, RGB approaches are most effective when paired with advanced image processing/ML or integrated with complementary modalities (e.g., thermal, fluorescence, or narrowband spectral cues). Accordingly, **Table 4b** recommends RF/SVM for small RGB datasets and CNN/Transformers when large labeled sets are available.

### 3.4 Thermal imaging or thermography

Thermal imaging derives its relevance directly from the stomatal and transpiration mechanisms described in Section 2. When plants experience stress, stomatal closure reduces transpiration and leads to elevated leaf surface temperatures. Thermal infrared sensing captures this temperature differential, offering a direct physiological indicator of water-related stress and energy balance. Thermography differs from other optical techniques by measuring emitted radiation instead of reflection (Mastrodimos et al., 2019). Thermal cameras detect infrared radiation and represent temperature values as false-color images, where each pixel corresponds to a specific thermal reading. Plant stress often leads to changes in leaf temperature, making thermography a useful method for early stress detection. For instance, when plants experience water deficiency, stomatal closure reduces transpiration and results in increased leaf surface temperature (Jones, 1999). Recent advances have improved thermal sensor sensitivity and integration with AI for better stress diagnosis (Garcia et al., 2024; Wang et al., 2025).

**Figure 6:**
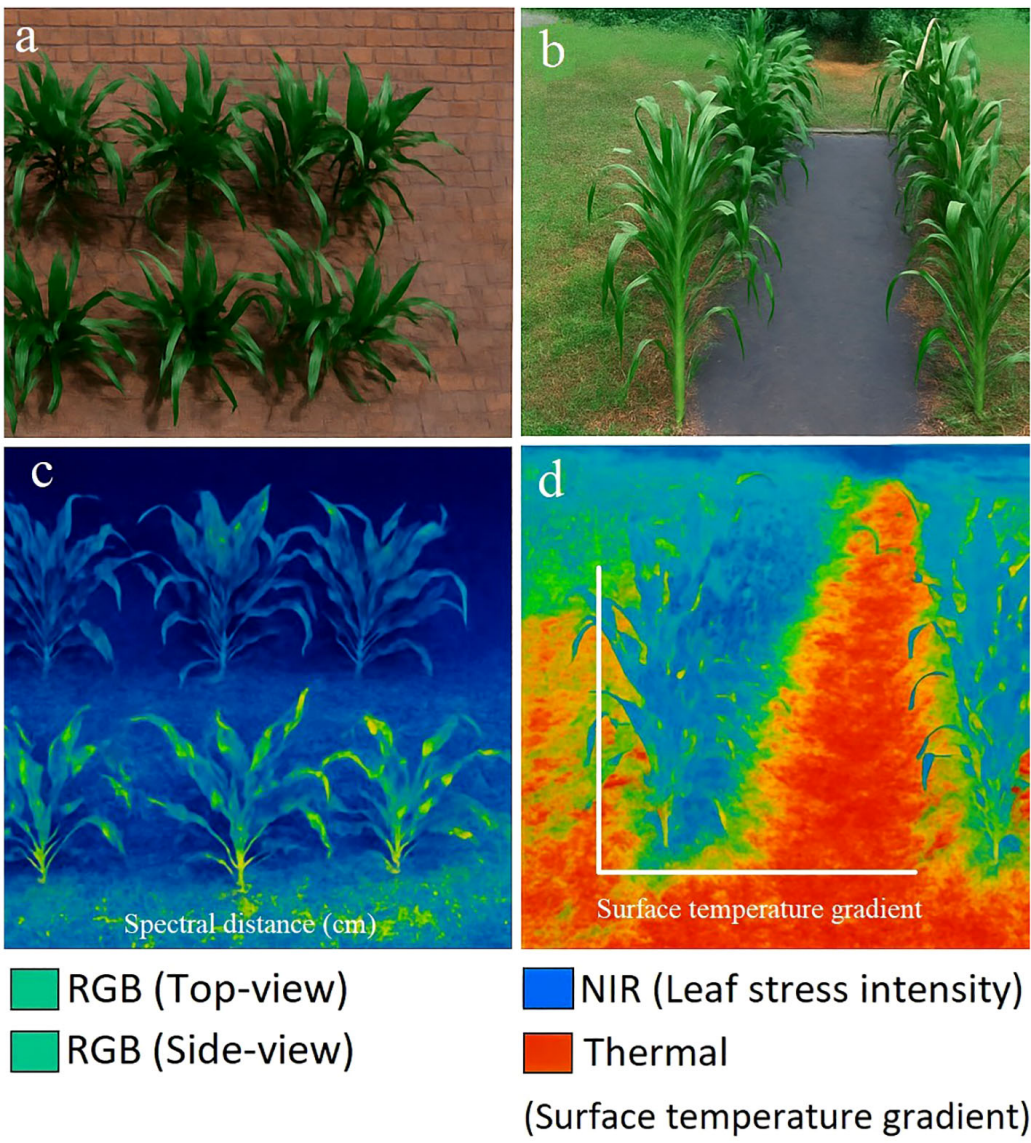
Multimodal imaging of maize under drought and well-watered conditions. **(a)** RGB top-view and **(b)** RGB side-view images show visible morphological differences between treatments. **(c)** Near-infrared (NIR) fluorescence image reveals variations in leaf stress intensity, while **(d)** the thermal image visualizes canopy surface temperature gradients (°C), with warmer colors indicating higher stress. Each panel includes scale bars and calibrated legends for quantitative comparison. Top row: well-watered; bottom: drought- stressed. Reprinted with permission from Casari et al. (2019).

This technique has been successfully applied to detect various plant stresses, including biotic factors such as *Aspergillus carbonarius* in grapes (Oerke et al., 2011), apple scab disease (Casari et al., 2019), and abiotic factors like drought stress in maize and sesame plants (Jones, 1999; Khorsandi et al., 2018). **Figure 6** illustrates how thermographic imaging uses thermal contrast to distinguish maize water conditions (Jones, 1999). Thermal contrast is a direct manifestation of the stomatal-closure/temperature response described in Section 2, yet it is not stress-specific; fusion with spectral/fluorescence cues (Section 3.7) and learned decision rules (Section 4) is typically required for attribution.

Thermography is considered a simple and rapid technology for plant stress identification. Portable thermal cameras, including smartphone attachments, have made the technology more accessible (Petrie et al., 2019). However, its performance is highly influenced by ambient environmental conditions, which restricts its reliability in field-based applications. Moreover, thermography provides limited specificity, as it cannot differentiate between stress types. As such, it is most effective when combined with complementary diagnostic methods to identify particular stressors or diseases with greater accuracy (Oerke et al., 2011; Khorsandi et al., 2018).

#### 3.4.1 Limitations and practical considerations

Leaf temperature signals are highly confounded by wind, humidity, irradiance, and canopy structure, reducing robustness in field conditions. While portable thermal cameras (including phone add-ons) are increasingly available, higher-resolution/high-sensitivity units remain moderately expensive. Critically, thermal contrast is not stress-specific; thermography alone cannot reliably distinguish which stressor is present, underscoring the need for multimodal fusion and contextual data.

### 3.5 Fluorescence spectroscopy

Fluorescence spectroscopy quantifies photosystem activity by measuring excitation–emission kinetics of chlorophyll and related pigments. Stress-induced perturbations alter fluorescence intensity and emission ratios, providing early biochemical indicators of photosynthetic efficiency and complementing reflectance- based sensing. Spectrophotometric imaging techniques rely on detecting attenuations of incident light across a broad range of wavelengths as it passes through plant leaves. However, the resulting images are often complex due to the presence of multiple pigments and compounds in plant tissues that exhibit overlapping spectral signatures (Lang et al., 1992). Fluorescence-based techniques provide a more selective alternative, as only a limited number of plant constituents fluoresce. These compounds absorb light at shorter wavelengths and emit at longer ones, allowing for clear separation between excitation and emission signals (Lang et al., 1992; Krause and Weis, 1984). Importantly, fluorescence imaging can reveal physiological changes such as decreased photosynthetic activity in response to pathogenic stress (Swarbrick et al., 2006).

Pulse-amplitude modulation (PAM) fluorescence is widely used to assess photosynthetic efficiency, employing pulsed and saturating light sources in combination with a continuous actinic light (Lawson and Vialet-Chabrand, 2018). This method enables the analysis of chlorophyll fluorescence kinetics, where a time-resolved signal reveals the impact of environmental stress on photosynthesis (Brooks and Niyogi, 2011). Since photosynthetic performance declines under stress, fluorescence signals increase due to energy dissipation. For accurate kinetic measurements, dark adaptation is required—typically for 30 minutes—to establish baseline fluorescence (minimum level) before excitation (Lei et al., 2016; Lichtenthaler and Rinderle, 1988; Murchie and Lawson, 2013). Light acclimation is also essential to ensure accurate and repeatable results (Gomes et al., 2012; Kalaji et al., 2014, Kalaji et al., 2018).

Fluorescence ratios derived from images and spectra are commonly used for evaluating plant stress. Ratios such as *F*_440_*/F*_690_, *F*_440_*/F*_740_, and *F*_690_*/F*_740_, especially under UV excitation (320–400 nm), serve as early indicators of plant health deterioration (Buschmann and Lichtenthaler, 1998). For instance, Burling et al., 2011 demonstrated how red/far-red and blue/green amplitude ratios distinguish between nitrogen deficiency and fungal diseases. Although well-established, these ratios can be refined through experimental optimization. Several fluorescence-sensitive indices in **Table 2** (e.g., PRI variants) operationalize these kinetics; protocol standardization (dark adaptation, excitation control) is critical for reproducibility.

**Figure 7:**
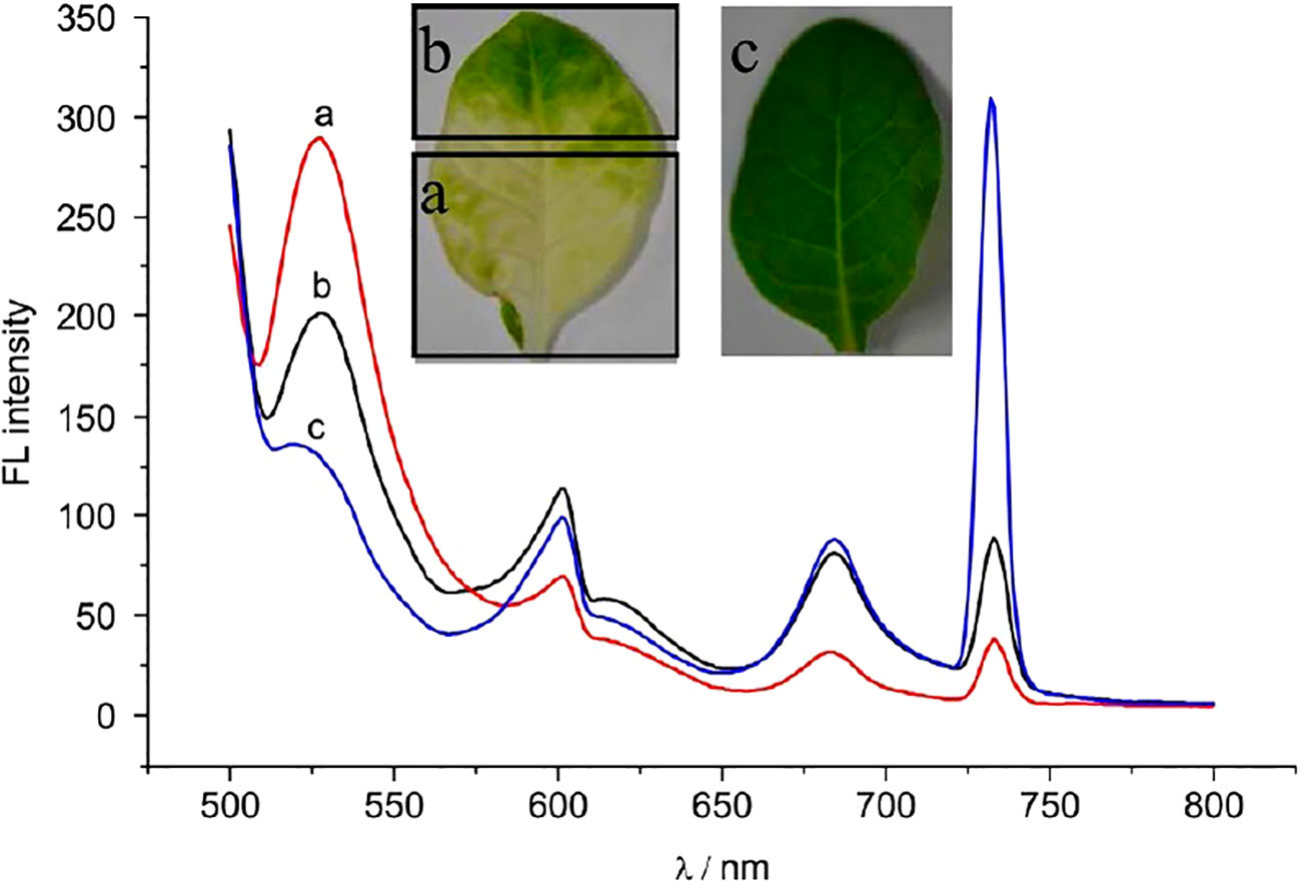
Fluorescence emission spectra of tobacco leaves under 488 nm excitation: **(a)** CMV-infected chlorosis, **(b)** CMV-infected with normal green coloration, and **(c)** healthy control (Lei et al., 2016).

Fluorescence spectroscopy also allows the localization and quantification of specific components within leaves. The fluorescence emission spectra of tobacco leaves under 488 nm excitation are presented in **Figure 7** (Lei et al., 2016).

This technique supports low-cost, handheld stress detection in various crops: drought stress in maize and passion fruit (Gomes et al., 2012), stress due to nutrient deficiency in maize and tomato (Kalaji et al., 2014), and citrus canker in grapefruit (Murchie and Lawson, 2013). While laser excitation improves sensitivity, the method is inherently non-selective, as fluorescence changes can arise from diverse causes. Therefore, for diagnostic specificity, fluorescence spectroscopy should be complemented with additional methods (Saleem et al., 2020). A common challenge is the time-dependent decline in fluorescence intensity during kinetic studies, though recent work by Saleem et al. (2020) mitigated this by rapidly analyzing spectra within 15 seconds of excitation.

#### 3.5.1 Limitations and practical considerations

Fluorescence methods offer high sensitivity to photosynthetic perturbations but often require dark adaptation or controlled illumination and can exhibit time-dependent signal decay. Diagnostic specificity is limited because fluorescence changes may arise from diverse stressors. Equipment spans from low- cost portable to laboratory-grade systems, creating cost and scalability challenges. Rapid-acquisition protocols mitigate some issues, but routine, large-scale field deployment still requires robust standardization. Combining fluorescence signals with RGB/thermal or narrowband spectral cues (Section 3.7) and ML models (Section 4) improves early detection and attribution.

### 3.6 Fluorescence imaging

**Figure 8:**
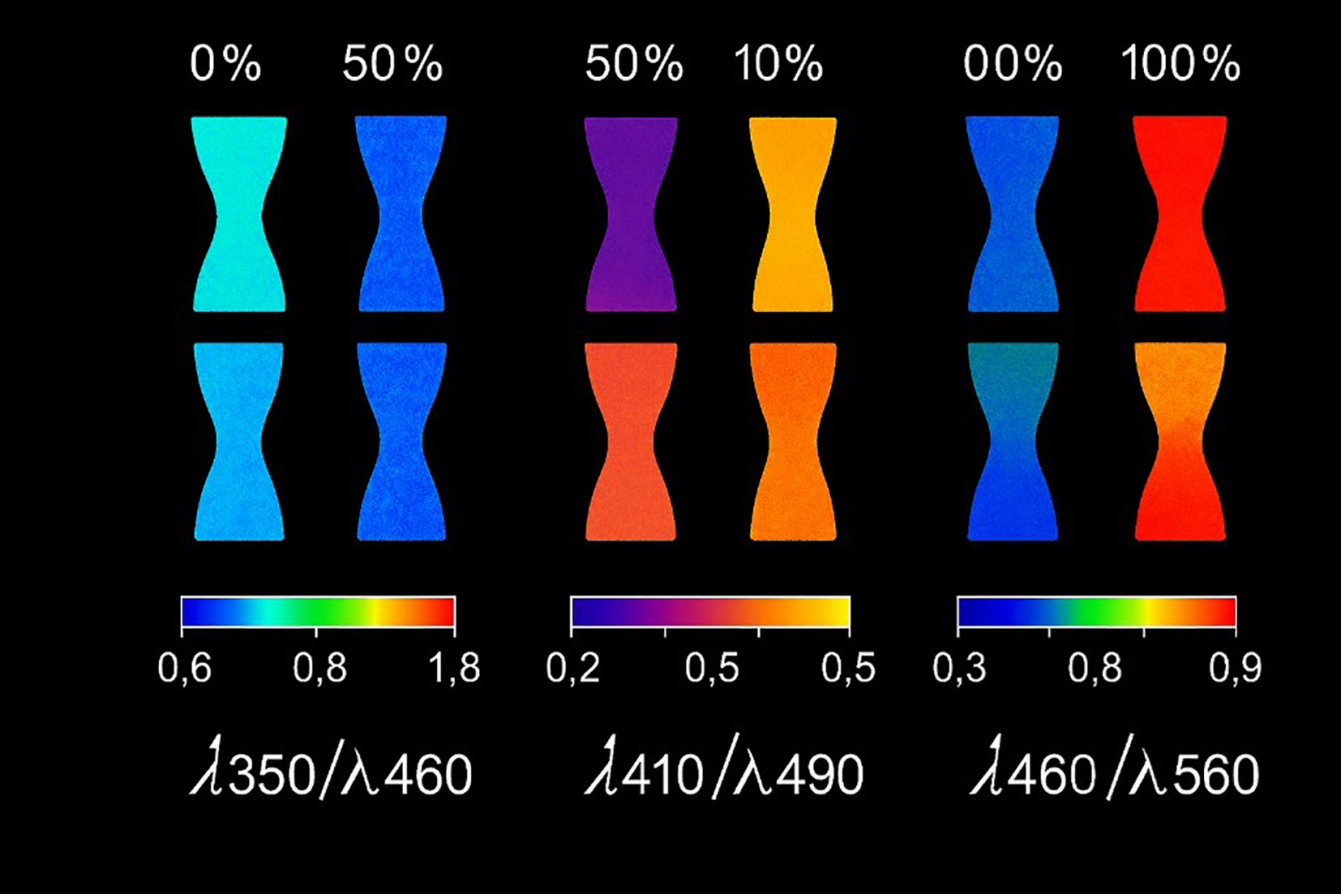
Ratios of fluorescence in barley leaves with increasing degrees of nitrogen deficiency. Reprinted from Konanz et al. (2014).

Fluorescence imaging captures spatially resolved emission patterns to visualize heterogeneity across leaves or canopies. By mapping changes in red and far-red fluorescence, it localizes stress responses linked to photosynthetic disruption and complements other modalities such as RGB and thermal imaging. Fluorescence imaging employs cameras to capture spatially resolved fluorescence emissions (**Figure 8**) and is often considered superior to fluorescence spectroscopy due to its capacity to acquire higher-dimensional data (Su et al., 2019). Unlike spectroscopy, which gathers a single spectral signature from a defined region, fluorescence imaging distinguishes between regions of interest and background by mapping spatial fluorescence intensity (Konanz et al., 2014). Multicolor fluorescence imaging, a subtype of continuous fluorescence imaging, utilizes ultraviolet excitation to record fluorescence emissions across several spectral bands, including blue (*F*_440_), green (*F*_520_), red (*F*_680_), and far-red (*F*_740_). These specific fluorescence bands are captured and combined to generate composite images (Konanz et al., 2014).

Multicolor fluorescence imaging, akin to multispectral imaging, enables the detection of various biotic and abiotic plant stresses. They have previously been applied in measuring herbicide stress in soybean ( (Li et al., 2017)), cold stress in tomato seedlings ( (Dong et al., 2019)), and grapevine, barley, and sugar beet stress ( (Su et al., 2019)). Moreover, there are low-cost and field-deployable solutions in the form of portable fluorescence imaging systems (such as smartphones with band-pass filters), which are not researched in the context of early detection of plant-level stress, yet might be applicable in practice ( (Chung et al., 2019)). Following earlier demonstrations, recent smartphone-controlled fluorescence modules with band-pass filtering and controlled UV excitation have been developed into field-deployable devices to measure early stress screening and spatial mapping of leaf scale ( (Garcia et al., 2025b)). Despite the rather low equipment cost of fluorescence-based methods ( (Konanz et al., 2014)), they may fail to distinguish between diseased and healthy tissue at the initial stages of infection. Hence, these methods should be complemented with other diagnostic tools to increase the early detection of diseases ( (Pe´rez-Bueno et al., 2019)). A big strength, though, is the sensitivity of fluorescence imaging, which can be effectively employed to detect as well as distinguish some of the stressors ( (Takayama and Nishina, 2009)). Relative to spectroscopy, imaging provides spatial context that downstream ML (Section 4) can exploit to separate lesions from the background, often improving early detection at a similar hardware cost.

#### 3.6.1 Limitations and practical considerations

Although equipment costs can be relatively low, fluorescence imaging can struggle at very early infection stages and is sensitive to excitation/emission setup and background fluorescence. Smartphone- based implementations are emerging, yet their robustness across diverse field conditions remains to be demonstrated. As with spectroscopy, combining fluorescence imaging with other sensing streams improves specificity and early detection.

### 3.7 Combination of sensors

To achieve a more comprehensive and reliable assessment of plant health, integrating multiple sensing methods is recommended rather than relying on a single technique. Several studies have demonstrated that plant stress detection benefits from the fusion of data acquired from diverse sensors (Moshou et al., 2011). Such sensor integration enhances diagnostic precision and robustness, reducing sensitivity to environmental variations (Moshou et al., 2011). However, the fusion of heterogeneous data streams presents considerable challenges, particularly in terms of harmonization and interpretation.

For example, Berdugo et al (Berdugo et al., 2014). used discriminant analysis in order to combine the data collected with the help of thermal imaging, hyperspectral analysis, and chlorophyll fluorescence ( (Berdugo et al., 2014)). This intermodal testing method allowed the accurate diagnosis of powdery mildew and differentiation comparison between cucumber plants infected with cucumber mosaic virus (CMV) and green mottle mosaic virus ( (Berdugo et al., 2014)). Complementary sensors have demonstrated some potential to enhance specificity and accuracy. However, the need to integrate large-scale data of various modalities with different data structures requires the additional elaboration of powerful methods of analysis and computation ( (Berdugo et al., 2014)). Machine learning (ML) has proven to be an effective approach to interpreting the high-dimensional data produced by the fusion of multiple modalities of sensorimotor measurements. The ML techniques have the ability to model nonlinear, complex relationships within the data and thus are an ideal fit for use when diagnosing plant stress. Though the use of different optical sensors has already been implemented to consider stress levels in agricultural crops ( (Adhikari et al., 2020; Bebronne et al., 2020; Brambilla, 2020; Banerjee et al., 2020; Cen et al., 2017; Raji et al., 2015)), applying advanced ML algorithms may positively affect their effectiveness to a large extent, which is further addressed in the following sections. In this review, we reference **Table 2** indices alongside modality choices to illustrate complementary sensitivity (e.g., SWIR water bands + thermal temperature + fluorescence PRI). These combinations directly inform the decision matrix in **Table 6** and classifier selection in Section 4.

### 3.8 Comparative assessment of monitoring approaches

To complement the fusion guidance in Section 3.7 and the decision matrix in Section 3.9, **Table 3** compares major optical modalities on practical criteria that drive field adoption: sensitivity/specificity to stress types, cost, mobility, calibration needs, and suitability for field conditions. This table is intended to be a one-look overview of the picking techniques within the bounds of reality; the full set of trade-offs and combinations of algorithms are explained in **Table 4b** and Section 3.10.

No single “best” modality exists; the choice depends on the earliness–specificity–cost balance (cf. **Tables 2**, **6**). Hyperspectral and fluorescence systems excel in detecting early physiological changes, while RGB and multispectral approaches are preferred for scalable coverage, and thermal imaging provides rapid water-stress flagging. Calibration governs transferability of radiometric controls (panels and illumination logs), emissivity and ambient corrections for thermal sensors, and dark-adaptation or excitation control for fluorescence, largely determining field reliability. In practical terms, low-cost deployments often use RGB/multispectral data with indices (**Table 2**) analyzed via RF or SVM models, whereas high-precision phenotyping favors hyperspectral imaging combined with CNN or Transformer architectures. Thermal imaging, when integrated with RGB or multispectral data, supports irrigation management and stress attribution (see Section 3.10 and **Table 6**).

### 3.9 Comparative decision matrix for sensor and algorithm selection

To support method selection under practical constraints, **Tables 4a** and **4b** critically compare imaging modalities and learning approaches across performance, cost/compute, portability, scalability, and best-fit scenarios. How to read **Tables 4a** and **4b**: Panel (a) aligns each modality’s physics with deployment constraints, while panel (b) pairs data characteristics with algorithm families.

Collectively, current imaging techniques reveal clear trade-offs. Hyperspectral systems provide the highest sensitivity and can detect subtle biochemical/structural changes, but are costly and computationally intensive. Multispectral approaches balance information content and affordability/portability, making them well-suited to targeted stress indices. RGB imaging is ubiquitous and low-cost (e.g., smartphones) but has limited diagnostic specificity and strong environmental dependence. Thermal imaging rapidly flags water-stress–related temperature changes yet cannot, by itself, disambiguate stress type. Fluorescence excels at photosynthetic efficiency assessment but often requires controlled conditions for reliability. Hence, sensor selection should match target application, resources, and precision needs, with multimodal fusion mitigating single-modality limitations.

Essentially, the same trade-offs apply among algorithms. SVMs and RFs are effective in spectral/small- scale and with more bit-compute and (when using the Random Forests model) feature importance, but they cannot adapt to the complicated structure of images. ANNs can effectively model nonlinearities and can be overfit without overregularization. Transformers and CNNs may achieve state-of-the-art image performance and learn spatial/long-range dependencies, but cannot learn without large labeled data, which is less interpretable. Ensembles/hybrids offer a compromise between a degree of precision in the peaks and large-scale power and medium interpretability. The amount/type of data should inform decisions,compute budgets, and intent (screening vs. decision support). This is a trade-off that we have made when preparing to introduce preprocessing and classifiers, as shown in **Table 4b** in Section 4, which we revisit when deploying in Section 5.

### 3.10 Synthesis design patterns

The trade-off between earliness and specificity remains a central consideration in sensor selection. Fluorescence and hyperspectral imaging detect early physiological shifts, while thermal imaging rapidly flags water stress but lacks specificity. RGB and multispectral systems, in contrast, excel in providing broader spatial coverage. The choice of modality, therefore, depends on where an application falls within the earliness–specificity–cost balance, as summarized in **Tables 2** and **6**.

The fusion ladder begins with late or decision fusion, which is most robust to missing data, and progresses to feature-level fusion when co-registration and illumination control are reliable. Data-level fusion should be applied only when alignment and acquisition are tightly standardized (Section 3.7; Section 5.2.1). Reporting of ablation studies and missing-modality resilience is strongly recommended to ensure transparency and comparability (Section 4.2.4).

In terms of cost and computational demand, comparable utility can be achieved through different pathways. One option is to combine RGB or multispectral data with vegetation indices (**Table 2**) and analyze them using RF or SVM for low-cost, low-compute applications. Alternatively, hyperspectral data can be processed using CNN or Transformer models, which offer higher sensitivity but at a greater computational cost. **Table 6** provides guidance for selecting along budget and performance contours, with escalation recommended only when additional sensitivity is operationally meaningful.

Regarding data requirements and performance, classical machine-learning models such as SVM and RF yield strong results when applied to small, well-curated spectral datasets. Deep learning approaches, on the other hand, become more advantageous when abundant labeled data or multimodal inputs are available (Section 4; **Table 6**). Robustness should remain a primary priority: cross-site and multi-year validation, illumination normalization, and detailed calibration logs usually have a greater impact on field transferability than model architecture alone (Section 4.2.4; Section 5.1).

Deployment strategies can be adapted to operational scale. Smallholder screening may rely on RGB or multispectral sensing paired with RF or SVM models and index-based inference (**Table 2**), optionally incorporating thermal data. Enterprise-level operations often employ UAV-mounted multispectral or thermal sensors with feature-level fusion integrated into decision support systems (Section 5.4). In research phenotyping, hyperspectral or fluorescence modalities combined with CNN or Transformer architectures are preferred for detailed mechanistic studies and index discovery.

Finally, consistent reporting is essential for reproducibility and comparison. Studies should document acquisition metadata (including sensor type, optics, and solar angle), calibration steps, split design to avoid data leakage, class-sensitive performance metrics and calibration measures, ablation and fusion strategies, and key operational indicators such as cost per hectare and time to alert. Collectively, these elements convert literature findings into a transferable design framework that supports robust, field-ready applications.

Each modality exhibits a distinct cost–sensitivity–specificity profile. Hyperspectral (HSI) offers high biochemical sensitivity and early detection but at a higher cost and lower throughput; multispectral balances sensitivity with lower cost and simpler deployment; RGB is the most accessible but less specific to subtle physiological changes; thermal is sensitive to stomatal conductance and water status but confounded by wind and radiation; chlorophyll fluorescence is highly specific to photosystem perturbations yet requires controlled excitation and calibration.

Smartphones introduce lighting variability, firmware-level processing, and device heterogeneity (auto white balance, sharpening, and tone mapping). To improve accuracy and comparability: (i) prefer RAW capture where possible; (ii) lock exposure, ISO, and white balance; (iii) include a reflectance/gray card or Spectralon panel in each frame for radiometric/color normalization; (iv) standardize capture distance and angle using simple jigs; (v) record ambient conditions (incident irradiance, wind, temperature); and post-process with device-specific color profiles. These steps reduce cross-device drift and improve transferability, especially when paired with transfer learning and domain adaptation (Patel et al., 2024a; Li et al., 2025a, Li et al., 2023).

This overview of sensor-specific trade-offs provides a practical foundation for understanding how multimodal datasets are integrated and analyzed using machine learning, which is discussed next.

## 4 Machine learning for multimodal sensor data analysis

**Figure 9:**
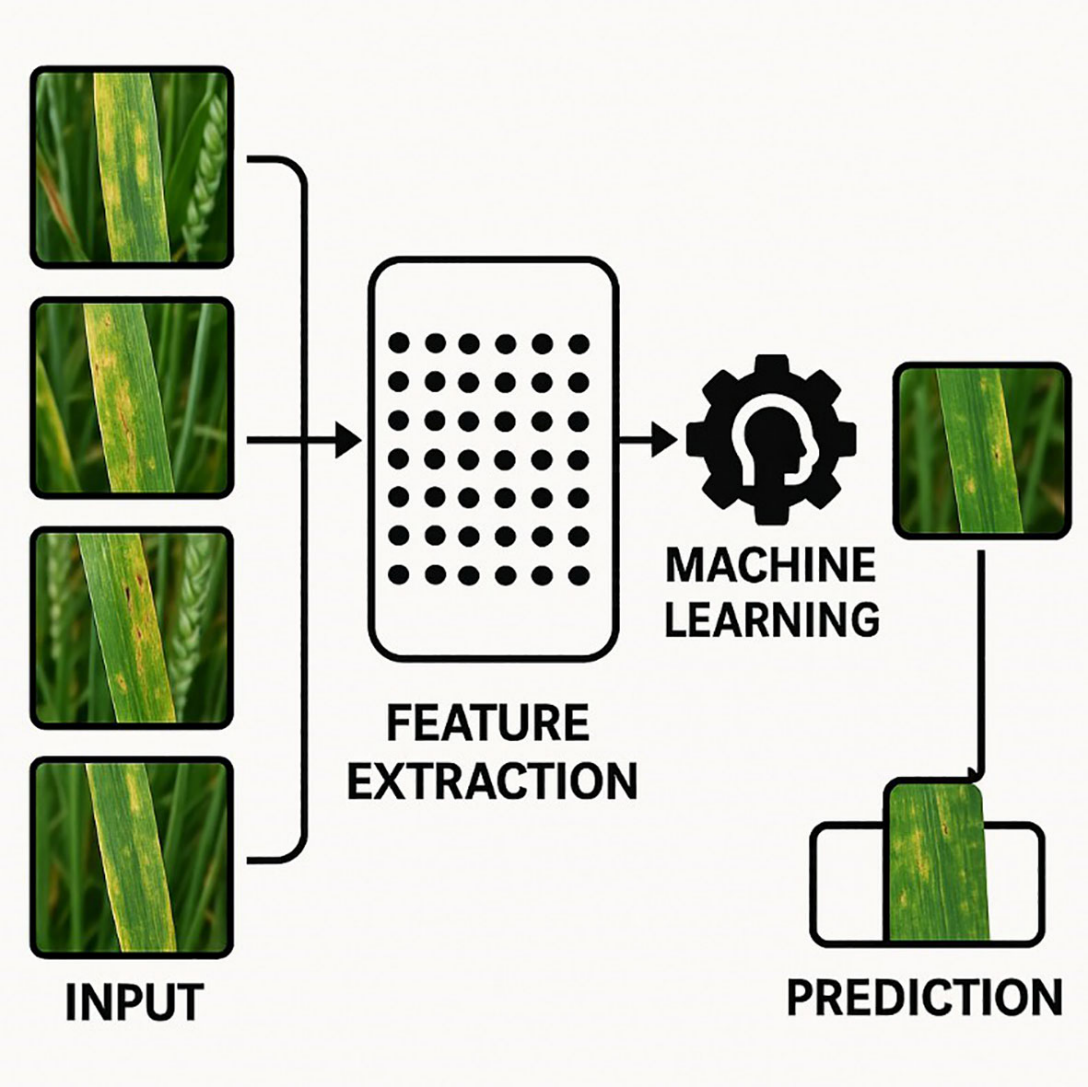
A basic pipeline for data analysis in machine learning, illustrating key phases such as data acquisition, feature extraction, and classification (Liakos et al., 2018).

Machine learning methods have opened new avenues for data processing across diverse fields such as medicine, environmental science, and economics. In its simplest form, machine learning utilizes algorithms that learn from existing data without requiring explicit programming instructions (Singh et al., 2016), enabling the detection of patterns that traditional analytical methods may overlook. The key steps in a machine learning approach typically include data collection and storage, feature extraction, classification, and pre-processing (Ramos-Giraldo et al., 2020). This section operationalizes the sensing choices in Section 3 by mapping data types and deployment constraints to algorithm families (see **Table 4**b) and by consolidating outcome evidence in **Table 6**. Where relevant, we reference indices from **Table 2** and design patterns from Section 3.10. A basic pipeline for data analysis in machine learning, including preprocessing, feature extraction, and model training, is shown in **Figure 9** (Liakos et al., 2018).

Machine learning approaches are increasingly being employed to analyze multimodal sensor data for plant stress detection, leveraging spectral and thermal cues across diverse crops and conditions.

Machine learning models, particularly ensemble and kernel-based techniques, have demonstrated strong capabilities in capturing nonlinear relationships between spectral features and environmental stress variables. These methods are increasingly being applied for drought prediction, nutrient optimization, and adaptive crop choice modeling in water-limited regions (Farooqui and Ritika, 2019; Adhikari et al., 2020; Banerjee et al., 2020).

SVMs and Random Forests are among the most widely used methods for spectral data classification because of their strong generalization ability with limited labeled data….

In agriculture, machine learning is particularly valuable due to its ability to uncover complex patterns by simultaneously analyzing multiple variables rather than treating traits individually (Singh et al., 2016). The plant environment is inherently complex, where various interacting factors play critical roles. Machine learning techniques help manage this complexity through processes such as classification, dimensionality reduction, and feature extraction. These uses are reflected in the study summaries and accuracies reported in **Table 6**.

Evaluating plant health using machine learning typically involves stress diagnosis, quantification, and discrimination. Identification refers to recognizing specific stressors and distinguishing their symptoms from others. Quantification allows for measuring the intensity of these stressors. Machine learning has been widely applied in these contexts, as summarized in **Table 6**. Use **Table 4b** as a quick “fit-for-purpose” map e.g., spectral vectors with limited labels → SVM/RF; high-resolution imaging or multimodal fusion.

CNN/Transformers (Section 3.10). Choosing the appropriate machine learning method varies with the specific problem, as no one approach fits every case. The sections below summarize key machine learning techniques and their use in processing agricultural data. For quick selection, **Table 4b** maps data regimes (e.g., small spectral vs. large image datasets) to suitable model families; we refer back to it in each subsection.

### 4.1 Preprocessing

**Figure 10:**
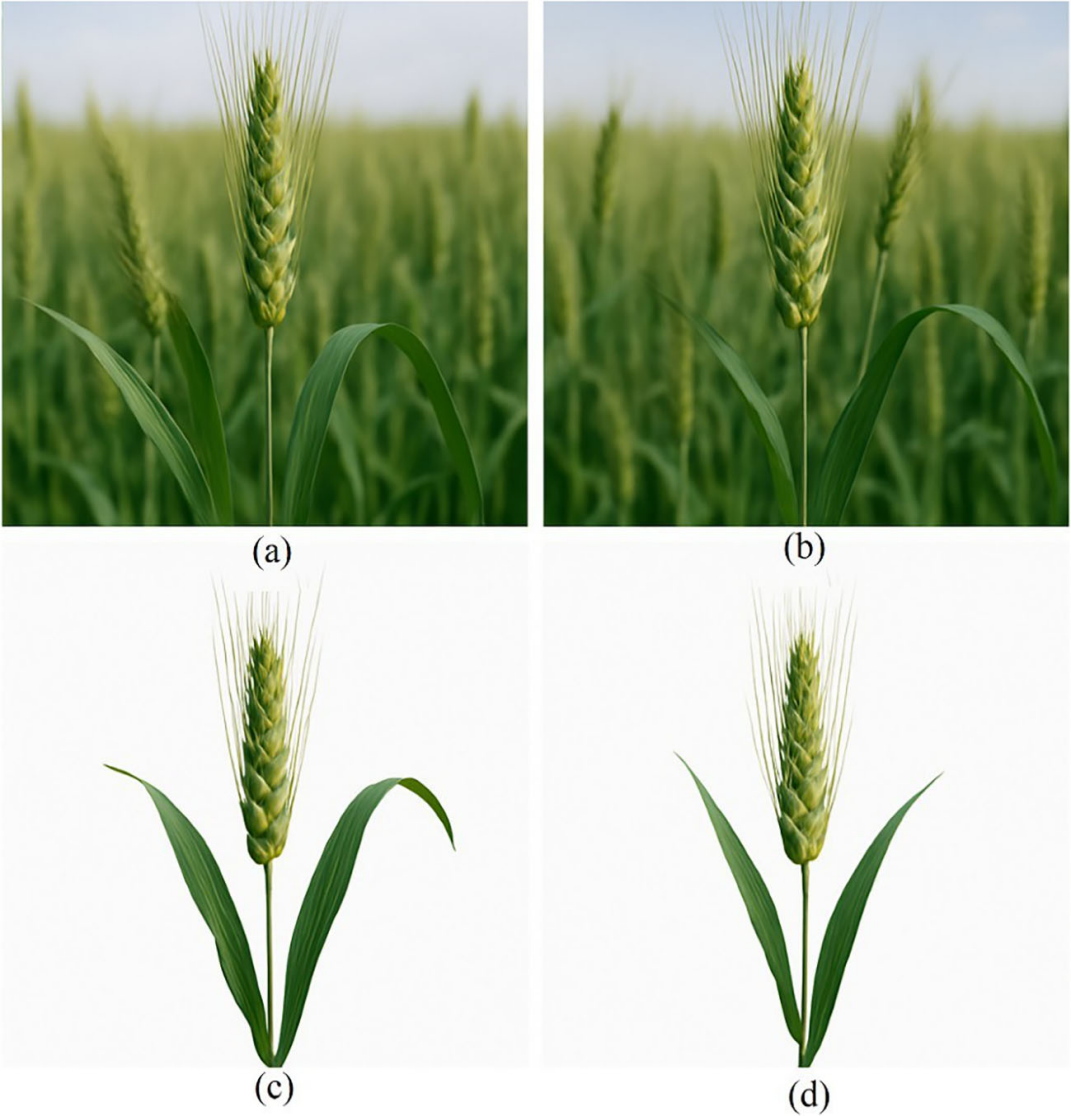
Image segmentation is visualized **(c, d)**. These are the original samples of both well-irrigated and drought-stricken wheat plants. In the first part, RGB values and a linear SVM were used to produce the initial segmentation images **(a, b)**, and then the mathematical morphology method was used to produce the denoised images **(c, d)** (Zhuang et al., 2017).

To ensure the accuracy and repeatability of classification outcomes, data preparation is essential (Tsaftaris et al., 2016). Pre-processing refers to a set of procedures aimed at enhancing the performance of classification algorithms by standardizing and making the input data more accessible and interpretable. In the context of image data, common preprocessing techniques include cropping images, removing backgrounds, enhancing contrast, applying thresholding, and reducing noise through filtering and clustering (Singh et al., 2016). The image segmentation process for well-irrigated and drought-stricken wheat plants is illustrated in **Figure 10** (Zhuang et al., 2017). Although the primary focus of this section is on imaging techniques, it is to be carefully noted that some techniques, such as Principal Component Analysis (PCA), may be applied to spectral data for dimensionality reduction and noise filtering. More recently, preprocessing pipelines combine classical spectral normalization (dark/white referencing, Savitzky–Golay smoothing, first/second derivatives, SNV/MSC) and band selection with deep feature learners (e.g., EfficientNet backbones or lightweight autoencoders) to improve feature extraction and boost plant disease detection accuracy; when labels are scarce, self-supervised pretraining and targeted data augmentation further stabilize downstream models (Patel et al., 2024a). Preprocessing choices should respect sensor properties from Section 3 (illumination control for fluorescence; radiometric normalization for hyperspectral; color constancy for RGB/multispectral) and also apply sensor-specific corrections, e.g., dark/white reference and vignetting/stray light correction; emissivity setting and ambient compensation for thermal; spectral smile/band alignment for hyperspectral; per-device color chart/ICC profiling and gamma linearization for RGB; and rigorous geometric co-registration for multimodal fusion while logging acquisition metadata (sensor, optics, solar angle, and irradiance) to ensure reproducibility.

#### 4.1.1 Color space conversion

**Figure 11:**
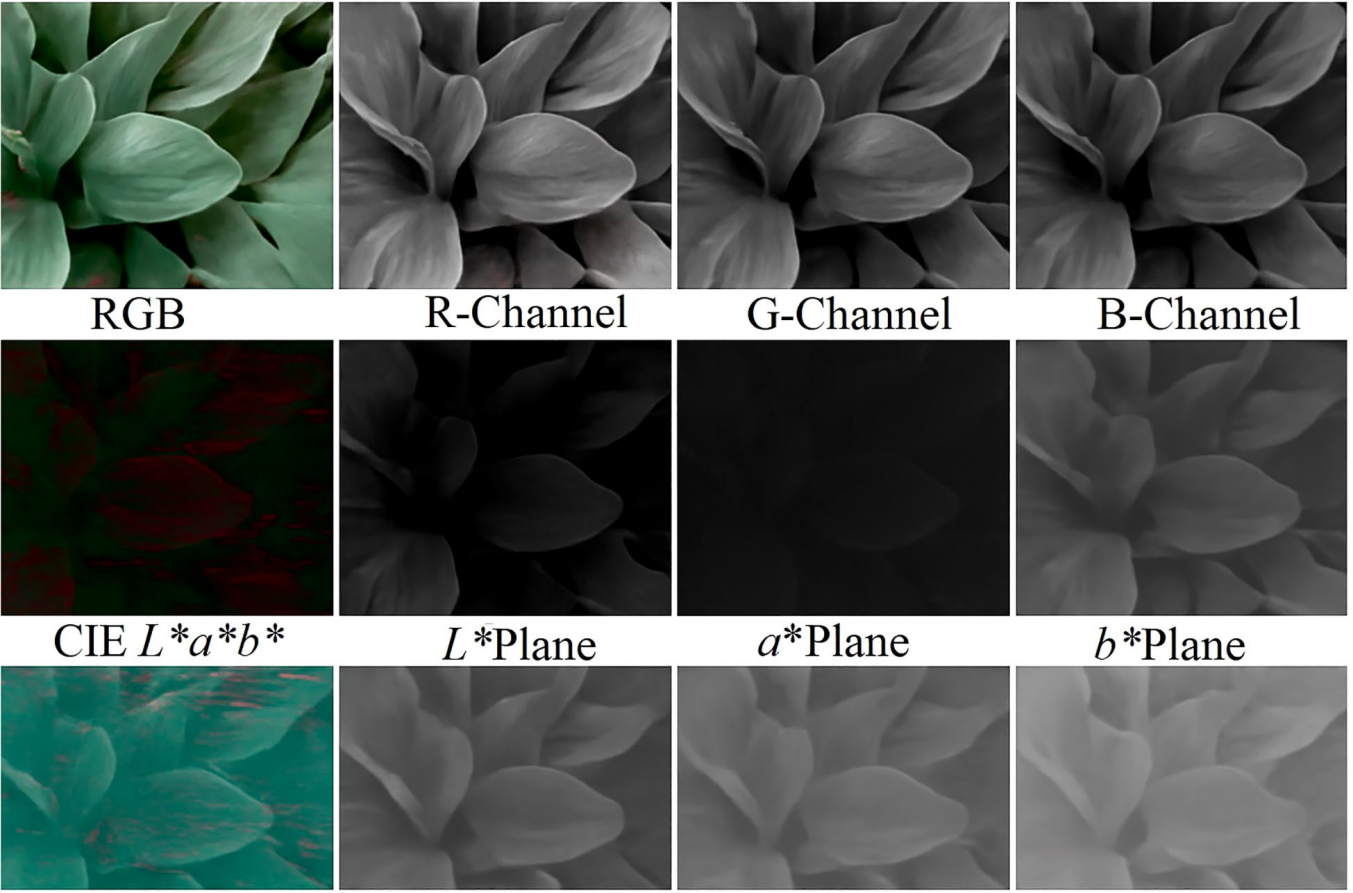
Other color space based on an RGB (color spectrum) image ( (Kahu et al., 2018).

In practice, CSC choices interact with the classifier families in **Table 4b**: hand-crafted color descriptors often pair well with SVM/RF on small datasets, whereas deep models can learn color invariances directly. Color space conversion (CSC) can be described as a data processing method to convert RGB images to other representations aimed at improving the analysis of the image. Color space derives more color attributes of pictures that are useful in feature-based image classification and feature extraction. CSC features have been extensively used in research done with RGB data to detect plant stress. Among them is the L*a*b color space (where L is the lightness number, a is the range between green and red, and b is the range between blue and yellow), which has previously been used to detect fruit rot, bacterial blight, and leaf and fruit spots in pomegranate plants ( (Dhakate and Ingole, 2015)). By the same token, the HSI (hue, saturation, intensity) color space has been found useful in detecting late scorch, early scorch, minute whiteness, cottony mold, and ashen mold in plants ( (Al Bashish et al., 2010)). It has also been applied in the detection of diseases in soybean using the color space of YCbCr (Y = luma component; Cb and Cr = blue and red difference chroma components) ( (Shrivastava et al., 2015)). Recent research has utilized advanced color space transformations to complement the disease detection accuracy of different crops ( (Wei et al., 2024)). The RGB-based color space representation used for image analysis is illustrated in **Figure 11** (Kahu et al., 2018). Practically, CSC decisions combine with the families of classifiers in **Table 4b**: hand-engineered color descriptors tend to be complementary with SVM/RF on small graphs, and deep models can learn to be color-invariant directly.

#### 4.1.2 Dimensionality reduction

Dimensionality reduction is a very important preprocessing method, which summarizes the data in a simpler form without giving up much of the information presented in the data. One of the most widely used methods for this purpose is Principal Component Analysis (PCA), which lowers data dimensions by projecting the dataset onto lower-dimensional subspaces that summarize the original features (Lever et al., 2017). PCA enables the integration of highly correlated variables into a single principal component, thus minimizing information loss. The initial principal component (PC1) explains the greatest variance, followed by PC2, PC3, and so forth, each being orthogonal to the preceding ones and capturing progressively smaller amounts of variance. These principal components (PCs) are often visualized in two-dimensional or three- dimensional PCA score plots, providing insight into data distribution and class separation. Selected PCs serve as inputs to ML models during preprocessing to improve their performance. PCA has been extensively used on both spectral and imaging data. Preferring an example (Lu et al., 2017a), used PCA to computer feature maps as a step in an image preprocessing pipeline ((Lu et al., 2017b)). Despite recent techniques developed to further optimize class separation, PCA remains an unbiased and widely used algorithm for reducing data dimensionality. One popular weakness with PCA, however, is that it is susceptible to outliers, which can affect the outcome components in a disproportional way ( (Wold et al., 1987)). Recent research has explored the concept of combining PCA with other dimensionality reduction algorithms to enhance feature extraction in multifaceted data sets ( (Lei et al., 2024)). On small spectral datasets, a common, computationally light recipe reflected in some entries of **Table 6** is PCA followed by SVM/RF (**Table 4**b).

#### 4.1.3 Segmentation

Image segmentation refers to the process of breaking down an image into meaningful parts, which may be the object of interest and the background of the image. Segmentation methods are also useful in the agricultural field because they minimize misclassifications and errors associated with background noise. The k-means clustering is a type of cluster approach that has been implemented successfully to recognize stress on plant images ( (Al Bashish et al., 2010)). Other methods might include pixel removal and masking to ensure proper disease detection in plants ( (Singh et al., 2016; Ma et al., 2018)). More recent developments see the introduction of deep learning-based segmentation models, like U-Net and YOLOv8-Seg, which have shown increased accuracy in outlining diseased areas in plant imagery ( (Ammar et al., 2024)). Segmentation has been shown to increase lesion-level measurement and decrease spurious correlations; this processing is indicated by increased precision/recall in the relevant rows of **Table 6**.

#### 4.1.4 Feature extraction

The task of feature extraction converts the raw data into a more convenient and useful representation understandable to machine learning algorithms ( (Tsaftaris et al., 2016)). This will allow the elimination of redundancy and selection of meaningful attributes of images. Typical methods are Local Binary Patterns (LBP) ( (Yue et al., 2011)), Color Coherence Vector (CCV) ( (Ojala et al., 2002)), and Global Color Histogram (GCH) ( (Vatamanu et al., 2013)), which are used to obtain sets of descriptive features without redundancy of information. Some examples of extracted features are color-related (channel variance) and texture (homogeneity and contrast) features ( (Ma et al., 2018)). After feature extraction, classification algorithms are used to analyze and identify the information and make appropriate decisions or categorize based on the learned patterns. Recent works utilized the latest feature extraction methods, such as deep learning models, to improve the accuracy of plant disease detection ( (Patel et al., 2024a)). Unlike deep feature extractors, features obtained via hand derivations can be fine-tuned to the modalities in **Table 1**, and either location can be found throughout the performance summaries in **Table 6**.

### 4.2 Machine learning approaches to classification

**Figure 12:**
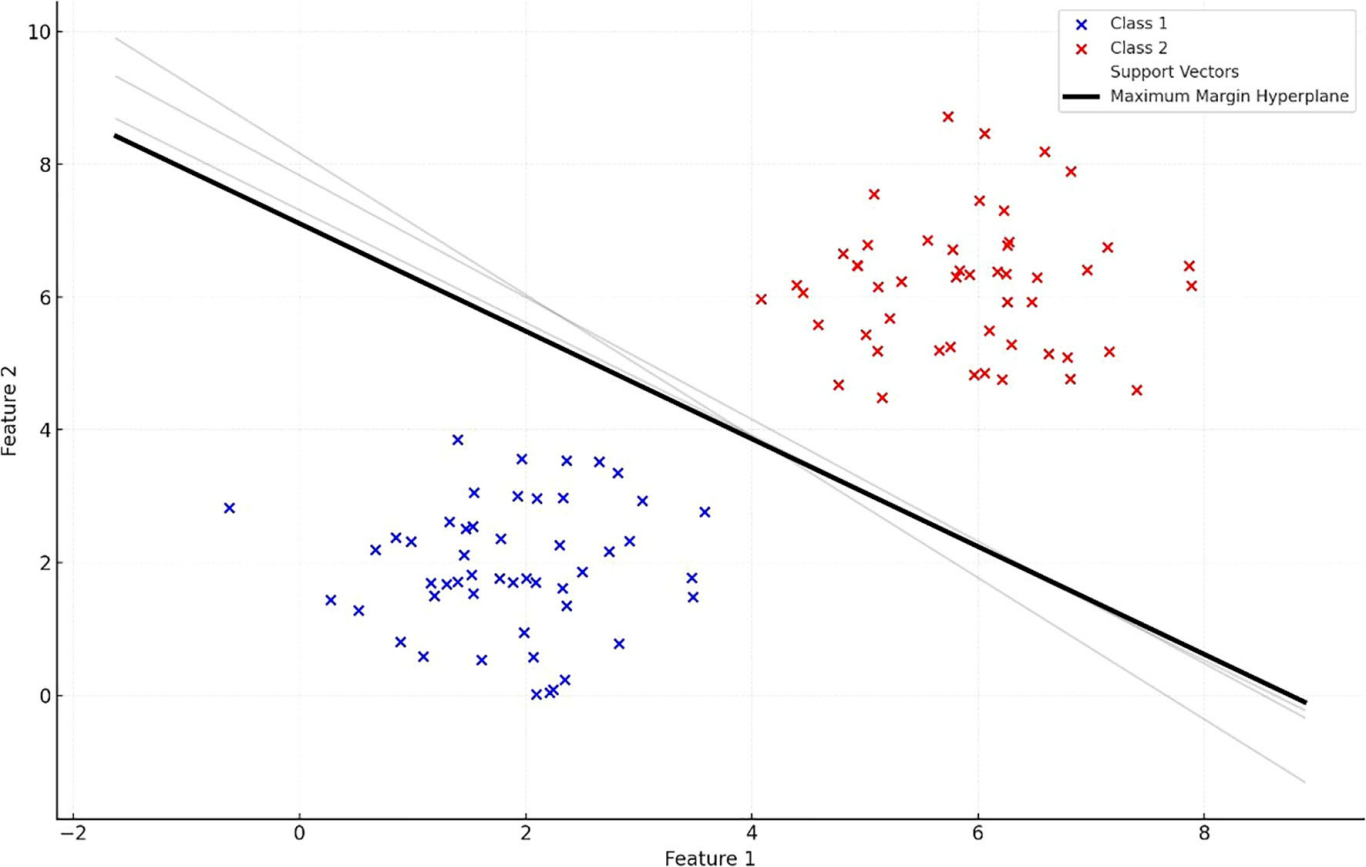
Examples of decision boundaries in other dimensions: 2D-dimension line, 3D-dimension plane, and higher-dimension hyperplane. These limits, which are usually defined through algorithms, such as support vector machines (SVMs), play a very important role in optimal class separation. Such techniques are used in agriculture to detect plant stresses, e.g., iron chlorosis in soybean and stripe rust or powdery mildew in wheat. SVMs can be effective but can fail in noisy datasets and areas with unclear boundaries, as well as where the boundaries between classes are not distinct ( (Naik et al., 2017; Xie et al., 2016; Cen et al., 2017)).

**Figure 13:**
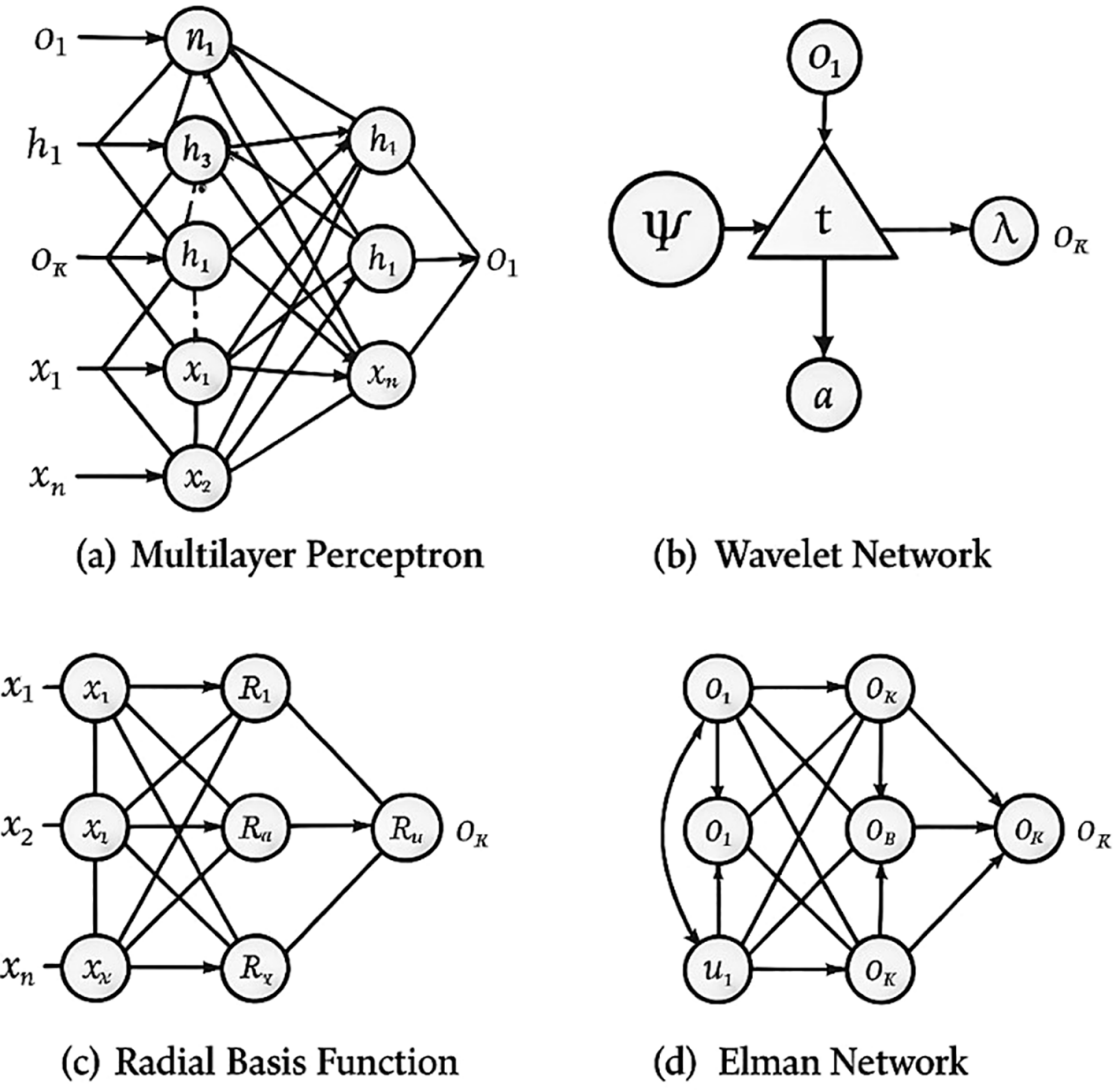
The four kinds of the Artificial Neural Networks (ANNs): **(a)** Multilayer Perceptron (MLP), which is a network with the input nodes (xi), hidden neurons (hi), output nodes (Oi), and weights (wi); **(b)** Wavelet Network, which is a network with a wavelet function (Psi), translation coefficient (ti), and dilation coefficient (la); **(c)** Radial Basis Function (RBF) Network, defined by radial basis functions (Ri); and **(d)** Elman Network, where recurrent connections are included between the hidden and the output layers and the context units (ui).

**Figure 14:**
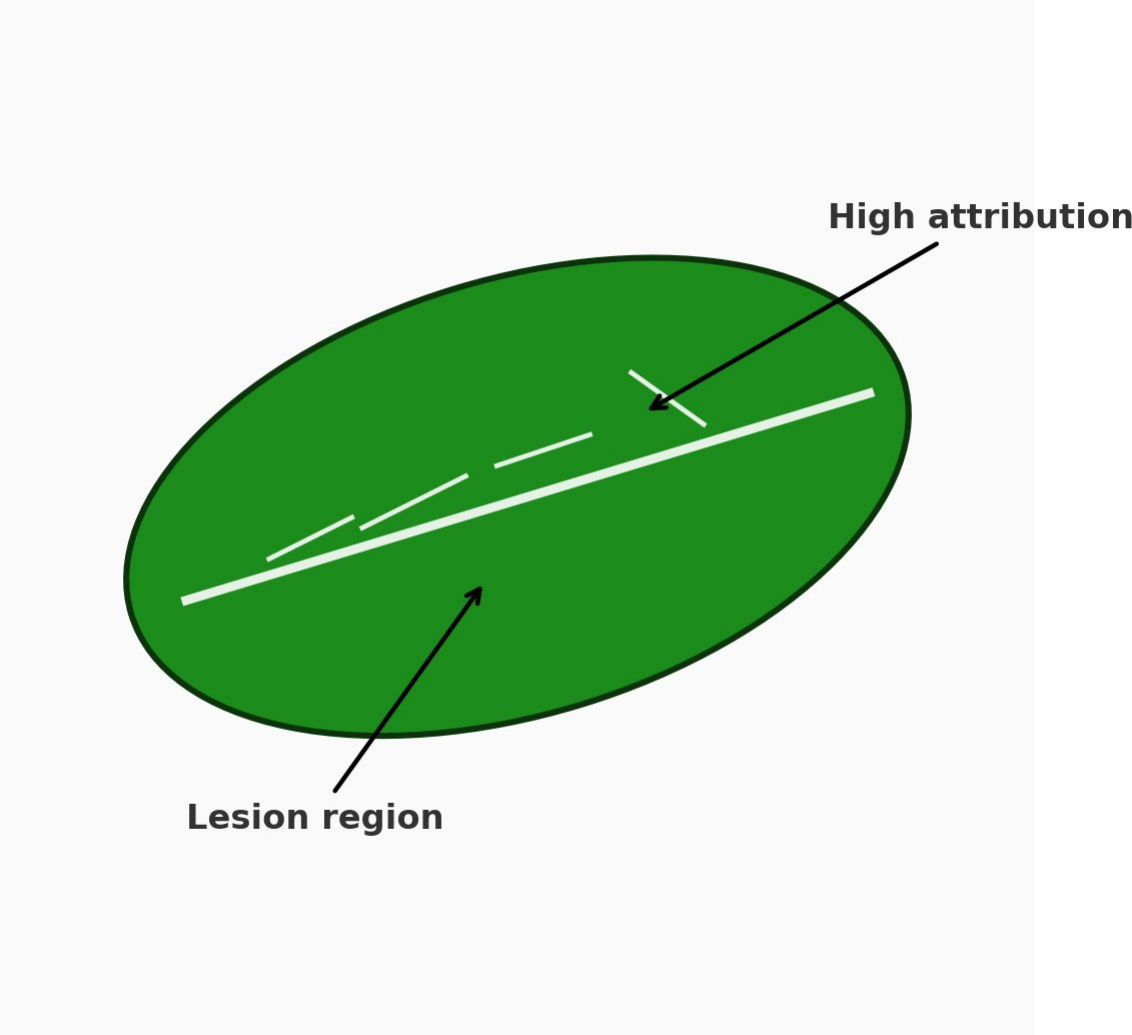
Example attention/attribution visualization (e.g., Grad-CAM) over diseased leaf regions to aid interpretability; ties to interpretability discussion in Section 4.2.4.

**Figure 15:**
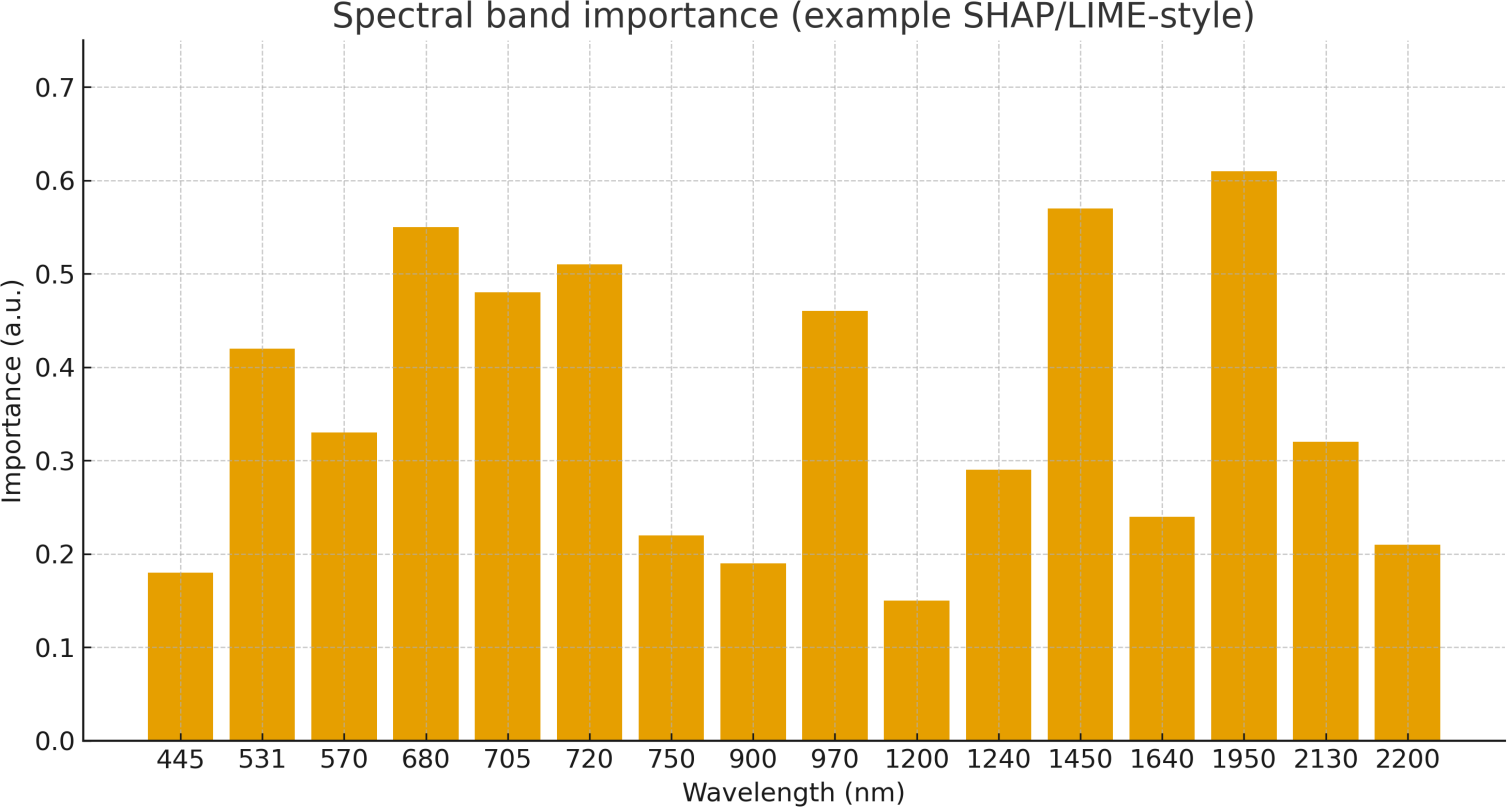
Example SHAP/LIME-style attribution for spectral features, indicating band importance for stress classification.

After the data preparation steps are completed, the dataset is passed to a machine learning technique for classification. These techniques identify patterns within the data to facilitate the categorization of previously unlabeled instances (e.g., stressed vs. healthy plants) (Rumpf et al., 2010). Machine learning techniques are broadly categorized into three types: supervised, unsupervised, and weakly supervised learning (Wang et al., 2017; Zhou, 2018). The primary distinction among these types lies in the nature of the input data: supervised learning requires labeled datasets for training; weakly supervised learning operates with limited labels, noisy labels, or coarse annotations; and unsupervised learning functions entirely on unlabeled data (Zhou, 2018). Clustering algorithms are a common example of unsupervised learning, where similar samples are grouped into clusters based on shared features (Rodriguez et al., 2019). Recent studies have explored self-supervised and hybrid approaches to better handle unstructured agricultural data (Huang et al., 2023). We illustrate linear vs. nonlinear decision boundaries and model families in **Figures 12**, **13**, and show attention/attribution examples in **Figures 14**, **15** to support interpretability (see Section 4.2.4).

Other classification methods that have been widely used in agriculture include Artificial Neural Networks (ANNs) (Dhakate and Ingole, 2015) and Support Vector Machines (SVMs). This study focuses on SVMs, ANNs, and deep learning as the tools to diagnose plant stress, but other algorithms also showed good results in the same domain, like the Random Forests ( (Rumpf et al., 2010)). Moreover, it is also believed that transformer models have become applicable to accomplish image classification tasks in precision agriculture, as they can be conveniently applied to feature extraction ( (Li et al., 2023)).Machine learning algorithms, regardless of their classification capabilities, are commonly affected by a variety of challenges, including overfitting, especially when training data is small, and computational complexity, especially when dealing with large volumes of image data. The association of these model families with typical data regimes, and the location of their reported accuracies, is given briefly in **Table 4b** and **Table 4b** respectively.

The latter can be solved by using methods of data augmentation, artificially increasing the quantity of training data by adding transformations like image rotation ( (Sladojevic et al., 2016)), color variation ( (Ghosal et al., 2018)), and mirroring ( (Esgario et al., 2020)). Data augmentation can be used to reduce overfitting besides enhancing the strength and generalization capability of the classification models. Notably, such augmentation steps need to be included before applying the classification algorithm in the data processing pipeline.

Though classical machine learning approaches such as the SVM, ANN, and the Random Forest have been found to be highly effective for detecting early-stage plant stress, their corresponding models typically apply hand-crafted features and their feature-processing pipelines to enable the delivery of high-quality results. They work best with SVMs: they achieve very good generalization performance on quite small training sets in high-dimensional spectral data, although they are not especially interpretable in terms of decision boundaries. Random Forests, by contrast, are computationally efficient, relatively resistant to overfitting, and provide feature importance measures that aid biological interpretability; however, they often underperform on highly complex multimodal datasets. ANNs offer flexibility in capturing nonlinear relationships but can suffer from instability and overfitting without careful regularization. In comparison, deep learning models such as CNNs and Transformers have demonstrated superior accuracy by automatically learning hierarchical feature representations directly from raw imaging and spectral inputs. CNNs are especially effective in spatial pattern recognition, while Transformer-based architectures show advantages in capturing long-range dependencies across multimodal datasets. These gains, however, come at the cost of requiring large, well-annotated datasets, high computational power, and reduced transparency compared to traditional models. Thus, the trade-off lies in balancing accuracy and scalability, where deep learning models dominate in large-scale, high-resolution applications, while classical ML models remain attractive in resource-limited or small-sample agricultural contexts. This trade-off framing aligns with the best-fit scenarios summarized in **Table 4b** and with the modality- and task-specific outcomes in **Table 6**.

#### 4.2.1 Support vector machine

Supervised learning methods, such as Support Vector Machines (SVMs), are widely used for classifying unknown data based on a labeled training dataset.

For instance, dimensionality reduction techniques like Principal Component Analysis (PCA) can be employed to reduce the majority of the training dataset (e.g., by more than 90%) to two principal components. These two components can then be visualized in a two-dimensional coordinate system using a PCA score plot. A decision boundary, often a line in two dimensions, can be built against the known classes (e.g., stress vs. healthy) to optimally divide up the data points into discrete categories. **Figure 12** demonstrates this idea ( (Cervantes et al., 2020)).

In three-dimensional data, the decision boundary is a plane; in data of higher dimension, it is a hyperplane. Although SVM is fundamentally a linear classifier, it can handle non-linear relationships in the data through the use of kernel functions, which allow the creation of non-linear decision boundaries. In multiclass classification tasks, multiple decision boundaries can be established to classify data into more than two categories. In a typical classification pipeline, once the decision boundary is generated during the training phase, it is used to categorize new, unseen test data. Despite the high-dimensional nature of raw data ranging from hundreds of dimensions in spectral data to millions in image data, dimensionality reduction often yields a compact set of principal components (e.g., 11 PCs) sufficient for effective classification. SVMs have been extensively applied in agricultural research (e.g., Naik et al., 2017; Cen et al., 2017; Xie et al., 2016). Consistent with **Table 4**b, SVMs fit well to small spectral features (small spectral features typically follow PCA) and small labels; **Table 6** lists some of these applications.

#### 4.2.2 Artificial neural network

An Artificial Neural Network (ANN) is a type of machine learning that tries to replicate the nature and functionality of a biological neural network ( (Krenker et al., 2011)).

The simplified structure consists of connected artificial neurons that receive many inputs and deliver an outcome after determining their weighted importance ( (Sladojevic et al., 2016)). ANNs have also been successfully utilized in different agricultural tasks to detect and categorize the plant stress factors. They have been applied, for example, to diagnose the crown rot in wheat ( (Humpal et al., 2020)), identify powdery mildew and soft rot in zucchini ( (Pineda et al., 2017)), classify biotic stress factors in pomegranate ( (Golhani et al., 2019)), and detect orange-spotted disease in oil palm trees ( (Dhakate and Ingole, 2015)). The benefits of ANNs are that they can be used without the need to have extensive knowledge or background in the domain or knowledge of how to interpret data. But they also have some drawbacks, such as overfitting and requiring a large amount of computational time ( (Tu, 1996)). ANN architecture has various categories that are applicable to various applications, as shown in **Figure 13** ( (Elsheikh et al., 2019)). **Table 6** shows that ANN baselines generally lag behind CNNs on large image tasks but are competitive in smaller tabular/spectral feature space tasks.

#### 4.2.3 Deep learning

Deep learning is a subfield of machine learning and applies ANNs to identify complex data relationships and can be defined as a network of layers; therefore, the term “deep” refers to the depth of the network. One of the most popular models that is used in agricultural practices is the Convolutional Neural Network (CNN) that performs convolution on the input data to classify the images ( (Jin et al., 2017)). CNNs and their variations have been broadly used to analyze plant stress, including tulip breaking virus ( (Polder et al., 2019b)), Potato virus Y ( (Polder et al., 2019a)), the extent of black rot in apples ( (Wang et al., 2017)), and biotic stress classification on cucumber leaves ( (Ma et al., 2018)), as well as coffee leaves ( (Esgario et al., 2020)). Recent advances in convolutional architectures such as U-Net and U-Net++ have significantly improved segmentation precision for leaf disease detection and canopy stress mapping (Farooqui et al., 2023; Saleem et al., 2020; Zhang et al., 2023b). The most commonly used popular pre-trained CNN models in such applications are VGG ( (Mohanty et al., 2016)), AlexNet ( (Zhang et al., 2018)), GoogLeNet ( (Ferentinos, 2018)), and ResNet ( (Ma et al., 2018)). Publicly accessible datasets, like the Diseased Wheat Database ( (Lu et al., 2017a)), or PlantVillage ( (Saleem et al., 2019)), are frequently used to train these models. One of the benefits of deep learning algorithms is that they can directly process raw data, which would not require any heavy preprocessing, like color space conversion, dimensionality reduction, segmentation, and feature extraction ( (Brahimi et al., 2017)). Moreover, some feature extraction is automatically carried out by deep learning models, which do not require *a priori* feature engineering ( (LeCun et al., 2015)). The primary shortcoming, though, is that it requires huge datasets (hundreds or even thousands of images) to gain high accuracy ( (Ferentinos, 2018; Dyrmann et al., 2016; Lu et al., 2017b)). Transformer-based architectures are increasingly explored for precision agriculture due to strengths in capturing long-range dependencies and multimodal fusion (Li et al., 2023). A concise side-by-side comparison of these algorithmic trade-offs is provided in **Table 4**, which pairs learning methods with recommended use cases and deployment constraints. When multimodal inputs are available (e.g., RGB+thermal+fluorescence/hyperspectral), Transformer-style fusion aligns with the “Large-scale multimodal sensing” use case in **Table 4**b and is reflected in higher- end results in **Table 6**, albeit with greater compute needs. Across algorithms, similar trade-offs emerge. SVMs and Random Forests are strong on spectral/small datasets, with lower compute and (for RF) feature importance aiding interpretability, but they struggle with complex image structure. ANNs offer flexible nonlinear modeling yet risk overfitting without careful regularization. CNNs and Transformers achieve state-of-the-art image performance and capture spatial/long-range dependencies but require large labeled data and high compute, and are often less interpretable. Ensembles/hybrids provide a middle ground, trading a bit of peak accuracy for robustness and moderate interpretability. Choice should be guided by data volume/type, compute budget, and intended use (screening vs. decision support).

#### 4.2.4 Critical considerations for ML models and benchmarking

Despite strong recent progress, several limitations constrain the reliability and transferability of machine learning models for plant stress detection.

##### 4.2.4.1 Data limitations and class imbalance

Most studies train on small, crop-specific datasets collected under controlled conditions, which restricts generalization across species, sites, seasons, and sensors. Class imbalance is common (healthy ≫ stress), biasing models toward majority classes and reducing sensitivity to rare stress conditions. Mitigations such as stratified sampling, cost-sensitive learning, focal loss, calibrated decision thresholds, and synthetic oversampling (with caution about distribution shift) remain underutilized. Transfer learning, domain adaptation, and active learning can reduce annotation burden and improve cross-domain robustness, but are unevenly adopted.

##### 4.2.4.2 Overfitting and evaluation leakage

ANN and deep learning models with large parameter spaces are especially prone to overfitting on limited or homogeneous data, yielding optimistic in-sample scores that degrade under real-world variability. Regularization (weight decay, dropout), extensive data augmentation, early stopping, and ensembling help, but evaluation design is critical: nested cross-validation, patient-/plot-/field-level splits that prevent leakage, and external validation across sites/years/sensors should be preferred over random splits.

##### 4.2.4.3 Interpretability and decision support

While CNNs and transformer models can reach high accuracy, their “black-box” nature limits trust and operational uptake. Explainable AI tools (e.g., SHAP, LIME, Grad-CAM) can expose which features, spectral bands, or image regions drive predictions, helping agronomists assess plausibility and revealing spurious correlations (e.g., background soil, tags). Because *post-hoc* explanations can be unstable, we emphasize sanity checks (e.g., perturbation tests), reporting of explanation variability, and linking attributions to physiological mechanisms when possible.

##### 4.2.4.4 Comparability and benchmarks

Cross-study comparison is impeded by heterogeneous datasets, preprocessing, and metrics. Community benchmarks with fixed train/validation/test splits, versioned releases, and standardized evaluation (accuracy along with precision/recall/F1, ROC-AUC, and PR- AUC for imbalanced data, and calibration metrics such as Brier score/reliability curves) would improve reproducibility. Shared datasets and challenge tasks akin to the visibility PlantVillage created for image classification are needed for multimodal sensing (RGB, thermal, fluorescence, hyperspectral) and cross- domain generalization (new fields/seasons/devices). Transparent reporting (confusion matrices, CIs), release of code and preprocessing pipelines, and model/data “cards” will further enhance comparability and accelerate progress toward robust, field-ready systems. In **Table 4**, we therefore report accuracy alongside class-sensitive metrics wherever available and note evaluation design to discourage optimism bias.

### 4.3 Validation, generalization, and evaluation protocols

Robust evaluation in plant-stress sensing requires a clear separation between (i) internal validation (e.g., *k*-fold cross-validation within a single dataset) and (ii) external testing on independent environments (different sites, seasons, cultivars, sensors). While internal validation estimates in-sample performance, external testing quantifies *real-world generalization* under domain shift (illumination, canopy structure, phenology, and background variability). We recommend reporting both, with site/species/sensor splits stated explicitly.

Use stratified *k*-fold cross-validation, ensuring no *leakage* (e.g., the same plant or plot does not appear across folds). When data are temporally correlated, adopt *blocked* or *grouped* CV.

Hold out at least one site or season as a *true external test set*. Report performance per site/species and aggregate with macro-averages to avoid dominance by large classes.

Plant-stress datasets are often skewed (rare early symptoms, majority healthy class). To counter this, use:

(1) *loss re-weighting* or *class weights*; (2) *resampling* (minority oversampling or majority undersampling); (3) *focal loss* for hard examples, and (4) *threshold tuning* using precision–recall curves. Always report the class distribution and the strategy used.

Accuracy can be misleading under imbalance. Report precision, recall, F1-score (macro/micro), PR-AUC, and confusion matrices (per class). For regression-like severity estimation, report *R*^2^, RMSE/MAE and Bland–Altman analysis for bias.

Transfer learning from large vision backbones and lightweight transformers improves data efficiency and robustness under cross-domain variability. Recent studies on transformer-based classification (Li et al., 2023), semi-supervised learning under limited labels (Huang et al., 2023), and efficient CNN/ViT variants for resource-constrained scenarios (Patel et al., 2024a; Debnath and Basu, 2025) demonstrate strong gains that translate to field settings. Practical enhancements such as improved color constancy, data augmentation, and normalization tailored to agricultural imagery further stabilize performance across devices and environments (Li et al., 2025a). We recommend explicitly evaluating models with (i) pretraining on generic plant/stress corpora, (ii) fine-tuning on target crops/sites, and (iii) *leave-one-site-out* protocols to quantify cross-site generalization. For field pipelines, semi-automated labeling and scalable annotation strategies also help close the domain gap (Tang et al., 2023b; Mi et al., 2020b).

## 5 Concluding remarks

Various optical sensors and algorithms have been employed to distinguish between biotic and abiotic stressors in plants, particularly diseases. While machine learning techniques are routinely applied to process imaging data—especially RGB images classical statistical methods are more commonly used in spectroscopic analysis. However, machine learning approaches are increasingly being integrated into spectroscopic data analysis, and this trend is expected to continue. To move beyond descriptive summaries, this review introduced a comparative decision matrix (**Tables 4a**, **4b**) that evaluates imaging modalities and machine-learning approaches across performance, cost, portability, and scalability, guiding selection of sensor–algorithm combinations to match resources and objectives. In this revised version, figures and tables are integrated in-line to support the narrative: **Figures 1**-**4** ground the spectral/hardware context, **Tables 1**, **2** consolidate wavelength targets and indices, and **Tables 3** connect sensing choices to algorithm selection and reported performance.

Many investigations in this area produce results that are applicable to only a limited range of plant species. Due to the significant variation in the reflectance properties of leaves among different plants, it is challenging to derive generalizable conclusions that are applicable to diverse crops and environments. In the future, plant stress detection is likely to move toward more universal outcomes rather than being species- specific. However, further research is needed to identify the traits and parameters that drive these results. The cross-species generalization gap highlighted here underpins the benchmarking and external-validation needs emphasized in Section 4.2.4 and summarized, where available, in **Table 6**.

Technologies such as smartphone-based Red-Green-Blue (RGB) imaging, fluorescence imaging, and thermography have the potential to scale to canopy-level analysis in both open environments and controlled settings. Recent advancements have improved both the quality and compactness of these systems. The optical resolution of modern smartphone cameras now rivals that of standalone digital cameras, with substantially improved sensitivity and on-device compute, enabling onboard analysis and decision support. Cloud computing and remote file management complement smartphones’ capabilities for heavier workloads. Optical zoom (often 2×–4×) further extends in-field utility. Nevertheless, distinguishing individual stresses, especially specific nutrient deficiencies, remains challenging: improved sensor sensitivity can increase susceptibility to environmental noise. Image segmentation and machine-learning pipelines that separate noise from targeted traits can mitigate this. Practically, this points to low-cost *RGB/multispectral + RF/SVM* baselines for broad coverage, with escalation to *hyperspectral + CNN/Transformers* in high-value or research settings (see **Table 4b**), and to targeted indices from **Table 2** for on-device screening.

### 5.1 Limitations and challenges

Despite notable progress, several obstacles remain before optical sensing and machine learning can be widely deployed in agricultural practice. Sensor-related factors such as high costs, calibration drift, and lack of standardized acquisition protocols hinder consistent results across systems. Environmental variability—including fluctuations in light, humidity, and temperature—introduces significant noise in field settings, reducing model robustness. Another persistent issue is reliance on small or imbalanced training datasets, which restricts generalization across species and stress conditions. While deep learning models can achieve high accuracy, they require large-scale annotated datasets and significant computational resources, which are not always feasible. Moreover, the “black-box” nature of many deep models raises interpretability concerns for agronomic decision-making. These constraints help explain variability across studies cataloged in **Table 6**; going forward, we advocate standardized reporting of splits, illumination controls, calibration logs, and class-sensitive metrics (precision/recall/F1, ROC-AUC/PR-AUC, and calibration) to improve comparability (Section 4.2.4).

### 5.2 Challenges for adoption and ethical considerations

#### 5.2.1 Data fusion challenges

Integrating hyperspectral, multispectral, thermal, fluorescence, and RGB data raises harmonization issues stemming from mismatched spatial resolution, spectral coverage, frame rate, illumination, and viewing geometry. Misregistration and radiometric drift can erode the gains from fusion. Practical mitigations include (i) co-registration using fiducials/UAV pose data; (ii) radiometric/illumination normalization (reference panels, per-session irradiance logs); (iii) standardized acquisition metadata (sensor, optics, altitude, solar angle); and (iv) architectures matched to conditions—early/data-level fusion when bands are well aligned; mid-level/feature fusion for mild misalignment; and late/decision fusion when modalities are heterogeneous or intermittently missing (see Section 3.7). Robust evaluation should report ablations, uncertainty calibration, and missing-modality resilience, aligning with Section 4.2.4. In deployment, start from indices in **Table 2** and escalate to richer fusion only when it consistently improves accuracy or earliness.

#### 5.2.2 Economic feasibility

High-end hyperspectral systems and large deep models offer sensitivity but carry significant capex (hardware) and opex (calibration, maintenance, labeling, and compute). Lower-cost options—smartphone + clip-on optics, handheld multispectral, or UAV RGB/multispectral—offer attractive total cost of ownership when paired with targeted indices and compact models (see **Table 4b**). A practical decision frame is:


$$\begin{array}{c} \text{NetBenefit}=(\text{Yiel}{\text{d}}_{\text{saved}}\times \text{Price})+\text{Inpu}{\text{t}}_{\text{savings}}-\frac{\text{Capex}}{\;}-\text{Opex}\\ -\text{Trainingtimevalue}, \end{array}$$

where *T* is the amortization horizon? Cooperatives and service models (e.g., sensing-as-a-service) can spread costs; edge/on-device inference curbs cloud expenses and connectivity dependence. Reporting cost per hectare, time-to-alert, and labor saved alongside accuracy is encouraged.

#### 5.2.3 Adoption barriers

Beyond affordability, uptake is constrained by training needs, usability, connectivity, and systems integration. Field-ready tools should provide (i) guided workflows (calibration prompts, quality checks);

(ii) localized interfaces and low-literacy modes; (iii) offline/edge operation with optional cloud sync; and (iv) interoperability with farm-management/DSS and IoT platforms. Extension programs and vendor maintenance plans are pivotal for sustained use. For smallholders, recommended on-ramps include RGB/multispectral + RF/SVM baselines (**Table 4b**), with escalation to hyperspectral + CNN/Transformer stacks in high-value crops or research settings (see Section 5.4).

#### 5.2.4 Regulatory and ethical considerations

AI-enabled diagnostics raise questions of data ownership, privacy, consent, security, and liability when recommendations drive irrigation or pesticide actions. Good practice includes:

- Governance: cleaR data-use policies; on-device processing by default where feasible; encrypted storage; opt-in data sharing; and, where privacy or connectivity is a concern, federated learning to update models without centralizing raw data.
- Transparency & accountability: human-in-the-loop for high-stakes actions; explanations/attributions that link model cues to physiological mechanisms (see Section 4.2.4); audit logs for recommendations and actions.
- Fair access: avoid widening the digital divide; support low-cost tiers, subsidies, or cooperative models; design for interoperability and open standards to prevent vendor lock-in.
- Environmental stewardship: consider device lifecycle (repairability, e-waste) and the energy footprint of training/inference; prefer lightweight models where possible.

### 5.3 Prioritized 5–10 year research roadmap

While current optical sensing and machine learning methods show strong promise, several gaps must be closed to achieve reliable, field-ready adoption. The roadmap below prioritizes near-term standardization and data efficiency, mid-term fusion and systems integration, and long-term scalability and equity.

Milestones should be benchmarked using the modality/algorithm pairings in **Tables 4a**, **4b**, the field scenarios in **Table 3**, and task-specific outcomes in **Table 6**.

#### 5.3.1 Short-term (1–3 years)

- Calibration & protocols: publish minimal acquisition and calibration checklists (illumination references, irradiance logs, emissivity settings, optics/altitude/solar geometry, and calibration audit trails). Provide reference image panels and open calibration scripts.
- Datasets & labels: release annotated, open-access datasets spanning species, sites, seasons, devices, and stressors; include standardized metadata (sensor, optics, flight height, time, weather). Encourage balanced splits for rare stress classes.
- Lightweight models: develop smartphone/edge-optimized models (quantization, pruning, distillation) with targets such as<20 MB model size,<200 ms on-device inference, and battery-friendly duty cycles.
- Evaluation hygiene: enforce leakage-safe validation (plot/field/year splits), external test sets, and report precision/recall/F1, ROC-AUC/PR-AUC, calibration (Brier, reliability curves), and time-to-alert. Include ablations, uncertainty calibration, and missing-modality robustness.

#### 5.3.2 Medium-term (3–5 years)

- Multimodal fusion frameworks: advance early/feature/decision-level fusion for hyperspectral, thermal, fluorescence, and RGB, matched to alignment and availability constraints (see Section 3). Provide reference pipelines and pretrained encoders.
- Explainable decision support: pair high-accuracy models with physiology-linked explanations (e.g., band/region attributions) suitable for agronomist review; standardize explanation stability checks and human-in-the-loop thresholds.
- IoT/DSS integration: connect sensing to decision support systems (DSS) and farm IoT (variable-rate spraying, fertigation), with audit logs, role-based access, and rollback options.
- Data-efficient learning: reduce label needs via transfer, self-/semi-supervised learning, active learning, and weak supervision; target ≥30% label reduction without accuracy loss across at least two new sites.
- Cost/performance reporting: alongside accuracy, report cost per hectare, labor saved, and operational uptime under field variability to support economic decisions.

#### 5.3.3 Long-term (5–10 years)

- Universal, cost-effective diagnostics: build models that generalize across species, geographies, and devices via domain adaptation and meta-learning; manage performance drift with continual learning and scheduled recalibration.
- Scaled deployment platforms: mature smartphone and UAV systems for large-area monitoring with edge inference, opportunistic cloud sync, and cooperative or service-based business models.
- Privacy-preserving ecosystems: favor on-device inference and federated learning so raw data stay local while models improve globally.
- Standards & governance: establish field-ready standards for data formats, APIs, and safety/regulatory compliance (data rights, liability), plus training and certification pathways for end users.
- Sustainability metrics: track lifecycle impacts (repairability, e-waste) and compute energy; prefer lightweight models and durable hardware.

#### 5.3.4 Benchmarking across the roadmap

At each phase, benchmark milestones using**Tables 4a**-**4b** (modality–algorithm pairings) aligned to the use-case tiers in **Table 3** (e.g., low-cost scouting vs. high-precision phenotyping) and summarize task outcomes using **Table 6**. Report not only accuracy but also calibration, time-to-alert, cost per hectare, and missing-modality resilience to ensure progress translates into robust, field-ready systems.

### 5.4 Practical applications

Translating sensing and analytics into day-to-day agronomy requires deployment pathways that are robust, affordable, and easy to use. Smartphone-integrated systems and low-cost portable sensors can democratize access to precision tools in resource-limited settings, enabling real-time disease surveillance, early warning for drought and nutrient stress, and site-specific management. Coupling diagnostics with decision support systems (DSS) and Internet of Things (IoT) platforms can close the loop from detection to action—for example, variable-rate spraying, fertigation/irrigation scheduling, and prioritized scouting based on risk maps. At the operations level, streamlined on-device inference (for offline use), minimal-step calibration workflows, and interoperable data standards (for integrating RGB, thermal, fluorescence, and spectral inputs) are critical for reliable adoption at scale. Partnerships among growers, extension services, and ag-tech vendors can accelerate diffusion by aligning user needs with platform capabilities and maintenance models. In operational terms, growers can begin with RGB/multispectral sensing plus RF/SVM (**Table 4b**) and indexed cues (**Table 2**) for rapid scouting, escalating to hyperspectral and Transformer/CNN models where precision or early biochemical sensitivity is needed; integrating thermal/fluorescence streams (Section 3.7) improves early-warning reliability.

The integration of advanced optical sensing modalities, including Red-Green-Blue (RGB), Near-Infrared (NIR), and Short-Wave Infrared (SWIR) imaging with machine learning, is moving plant stress detection from species-specific experiments toward scalable, field-ready decision tools. While constraints in cost, calibration, environmental variability, and data availability persist, converging advances in multimodal fusion, explainable AI, domain adaptation, and smartphone-based platforms provide a practical roadmap. Looking ahead, the next stage of progress will depend on addressing several critical challenges: (i) establishing standardized calibration frameworks for multimodal data fusion to ensure cross-sensor consistency; (ii) expanding open-access, annotated multimodal datasets that capture cross-species and cross-environment variability; and (iii) developing field-ready standardization protocols that harmonize acquisition, preprocessing, and benchmarking across studies. Overcoming these gaps will be essential for robust model generalization and large-scale deployment. For a concise side-by-side view of current trade-offs, see **Tables 4a**, **4b**. Embedding these capabilities within DSS/IoT ecosystems will link sensing to real-time, actionable decisions, paving the way for reliable, interpretable, and equitable stress-detection systems that support sustainable agriculture under increasing climatic and economic pressures. By using **Figures 1**–**4** and **Tables 1**–**6** as a coherent scaffold linking physics, sensors, indices, models, and outcomes, this review outlines a transparent, end-to-end path from spectral mechanisms to actionable interventions in precision agriculture.

## Data Availability

The original contributions presented in the study are included in the article/supplementary material. Further inquiries can be directed to the corresponding author.
